# A Biomimetic Dual‐Targeting Nano‐APA‐Editor Reprograms the 3'UTR Landscape for Tongue Squamous Cell Carcinoma Therapy

**DOI:** 10.1002/advs.76126

**Published:** 2026-06-15

**Authors:** Yiran Ao, Bin Gu, Hui Zhao, Kun Tan, Qin Zhao, Zhengjun Shang

**Affiliations:** ^1^ State Key Laboratory of Oral & Maxillofacial Reconstruction and Regeneration Hubei Key Laboratory of Stomatology Key Laboratory of Oral Biomedicine Ministry of Education School & Hospital of Stomatology Wuhan University Wuhan P. R. China; ^2^ Taikang Center for Life and Medical Sciences Wuhan University Wuhan P. R. China

**Keywords:** 3'UTR reprogramming, alternative polyadenylation, CRISPR/Cas9, NUDT21, oral squamous cell carcinoma

## Abstract

Targeting post‐transcriptional dysregulation of tumor suppressors represents a new frontier in cancer therapy. Here, we identify the alternative polyadenylation (APA) regulator NUDT21 as a pivotal therapeutic target in oral squamous cell carcinoma (OSCC). NUDT21 is highly upregulated, correlating strongly with poor survival and advanced clinical stage. We outline a pathogenic mechanism whereby NUDT21 drives this phenotype by forcing a network of tumor suppressor transcripts, notably PTEN, into translationally‐repressed, long‐3'UTR isoforms. To therapeutically “re‐engineer” this APA switch, we design a “Nano‐APA‐editor.” This platform features an HMSN core with an sgRNA‐NUDT21 payload and a hierarchical targeting strategy: a cancer‐educated dendritic cell (DC) membrane for biomimetic camouflage and homotypic affinity, “gated” by a TA‐aptamer for final precision. This system enables potent and selective NUDT21 silencing, driving a shift toward short‐3'UTR isoforms. Consequently, the Nano‐APA‐editor effectively reinstates PTEN and associated suppressors and inhibits multiple malignant phenotypes in vitro. In an orthotopic OSCC model, it demonstrates profound tumor regression, outperforming conventional chemotherapy (PTX) with excellent biocompatibility. In vivo analysis confirmed target engagement (NUDT21‐down) and functional restoration (PTEN‐, WEE1‐, TGF‐β‐up). This work validates a “post‐transcriptional re‐engineering” strategy, executed by a logically designed nanoplatform, as a powerful and safe modality for precision gene therapy.

## Graphical abstract

This study unveils a pathogenic axis in oral cancer where NUDT21 suppresses a tumor suppressor network, prominently PTEN, via 3'UTR lengthening. To exploit this transcriptomic vulnerability, we developed a biomimetic Nano‐APA‐editor. By delivering CRISPR/Cas9, we reprogrammed the global 3'UTR landscape, restoring multi‐target tumor suppression and inhibiting growth. Our findings establish post‐transcriptional 3'UTR re‐engineering as a viable precision nanomedicine paradigm.

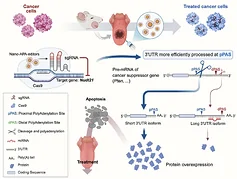

## 1 Introduction

Head and neck squamous cell carcinoma (HNSC) remains a formidable global health challenge, with oral squamous cell carcinoma (OSCC) representing its most prevalent and insidious subtype [1, 2]. Despite multimodal therapeutic strategies encompassing surgery, radiotherapy, and systemic chemotherapy, the 5‐year overall survival rate for HNSC patients has stagnated, largely due to high rates of locoregional recurrence, distant metastasis, and profound therapeutic resistance [3, 4]. The clinical utility of conventional chemotherapeutics, such as paclitaxel (PTX), is further constrained by severe dose‐limiting systemic toxicities and acquired resistance [5, 6]. This grim clinical landscape underscores an urgent and unmet need to identify novel molecular targets that underpin OSCC pathogenesis and to develop precision nanomedicines capable of exploiting these vulnerabilities with high specificity and minimal off‐tumor effects.

Emerging evidence illuminates the dysregulation of post‐transcriptional gene control as a pivotal, non‐mutagenic driver of tumorigenesis [7, 8]. Alternative polyadenylation (APA), a ubiquitous mechanism that generates distinct mRNA isoforms with variable 3' untranslated regions (3'UTRs), is increasingly recognized as a critical layer of this regulatory network [9, 10]. The 3'UTR landscape is paramount to cellular fate, as these regions harbor cis‐regulatory elements, most notably microRNA (miRNA) binding sites, that dictate mRNA stability, translational efficiency, and subcellular localization [11, 12]. A global reprogramming of the APA landscape, which alters the canonical 3'UTR‐mediated suppression of key oncogenes or tumor suppressors, has been identified as a new hallmark of cancer, profoundly impacting cellular proliferation, metabolic reprogramming, and invasive potential [13].

Central to the fidelity of APA site selection is the cleavage factor I (CFIm) complex and its core component, NUDT21 (CFIm25) [13, 14]. NUDT21 typically functions to promote the utilization of distal polyadenylation sites (dPAS) [15]. Our investigation into the HNSC cohort of The Cancer Genome Atlas (TCGA) revealed that *NUDT21* is significantly upregulated in tumor tissues relative to adjacent normal tissues. Strikingly, elevated *NUDT21* expression correlates unequivocally with advanced clinical stage and serves as a powerful independent predictor of dismal overall survival. This evidence strongly implicates *NUDT21* as a pivotal prognostic biomarker and a non‐oncogene addiction target in OSCC, suggesting that its therapeutic suppression could reactivate dormant tumor‐suppressive pathways.

**SCHEME 1:**
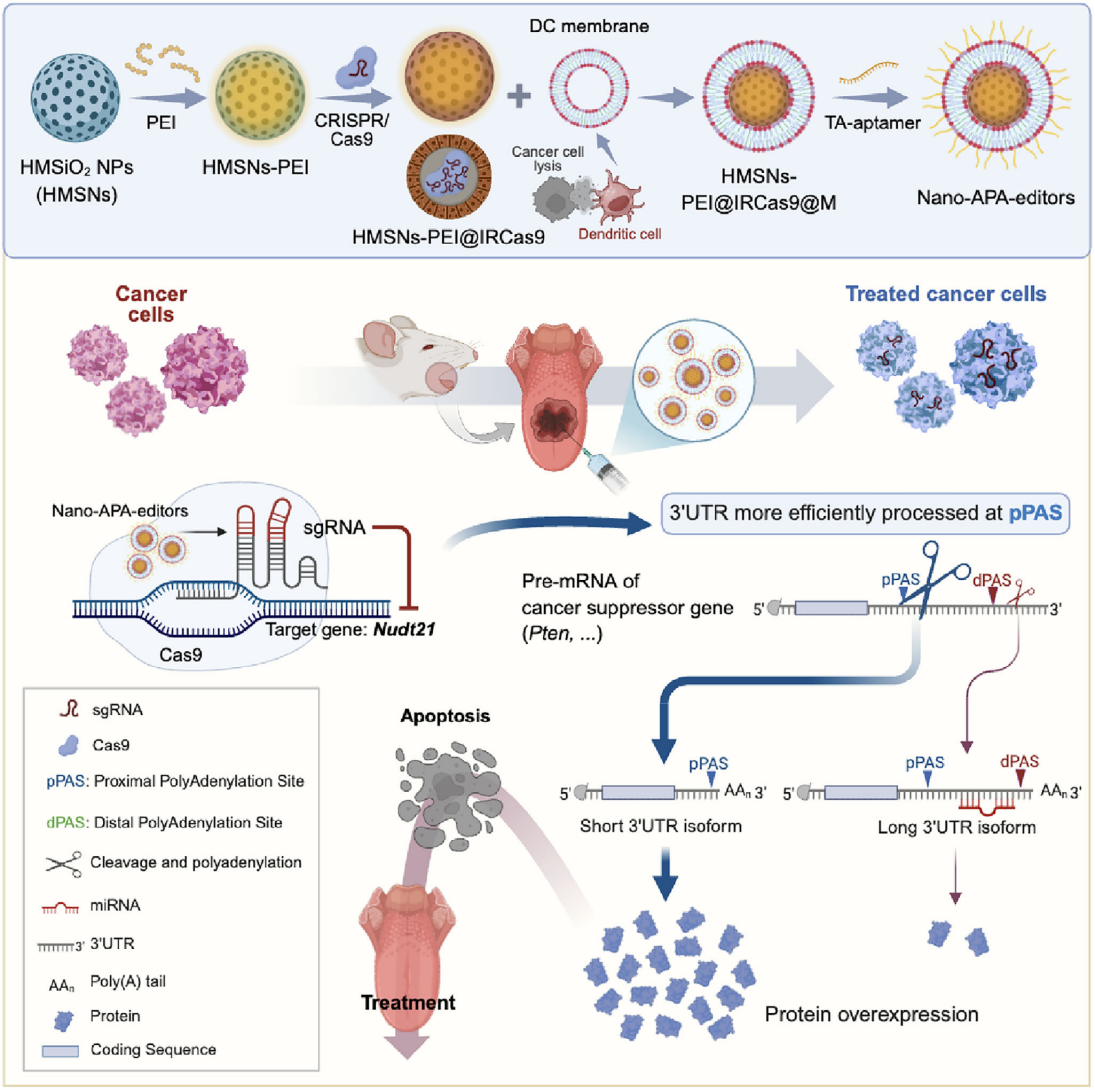
Schematic diagram of the design and function of Nano‐APA‐editors.

Despite the profound therapeutic potential of targeting *NUDT21*, the safe and efficient in vivo delivery of gene‐editing payloads, such as CRISPR/Cas9 systems, to solid tumors remains a formidable barrier. To circumvent challenges of enzymatic degradation, immune clearance, and non‐specific biodistribution, we engineered a biomimetic, dual‐targeting delivery platform termed “Nano‐APA‐editors.” This nanoplatform features a hollow mesoporous silica nanoparticle (HMSN) core, functionalized with polyethyleneimine (PEI) to electrostatically complex and protect CRISPR/Cas9 (with sgRNA) targeting *NUDT21*. Critically, the nanoparticle is camouflaged with a dendritic cell (DC) membrane, pre‐processed with cancer cell lysates, to bestow homologous targeting capabilities and immune evasion. This biomimetic vesicle is further functionalized with a cancer‐specific TA‐aptamer, achieving a dual‐receptor‐mediated targeting paradigm (Scheme 1). Herein, we demonstrate that this Nano‐APA‐editor precisely targets OSCC cells, effectively silences *NUDT21*, and therapeutically rewires the APA landscape, driving widespread 3'UTR shortening across a network of tumor suppressors. Specifically, we identify the translational derepression of PTEN and the subsequent inhibition of the PI3K‐Akt pathway as a critical molecular hallmark of this response, culminating in profound and targeted tumor regression in an orthotopic OSCC model that surpasses the efficacy of conventional chemotherapy.

## 2 Results and Discussion

### 2.1 *NUDT21* is Upregulated in HNSC, Correlates With Poor Prognosis, and is Associated With Tumor Suppressor mRNA Levels

**FIGURE 1:**
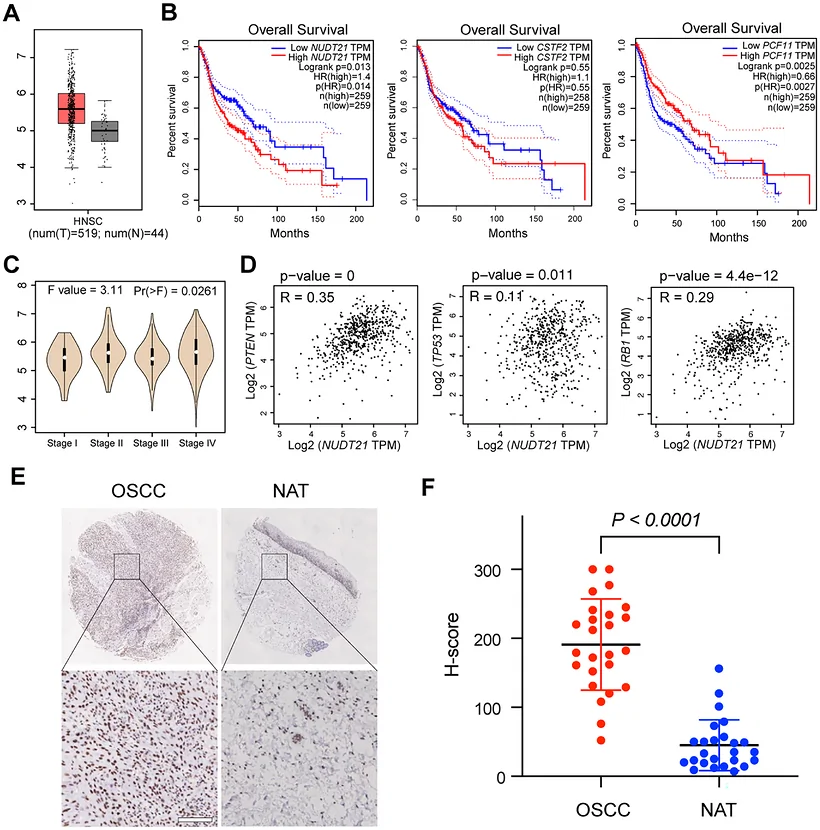
The APA factor NUDT21 is highly associated with the occurrence and poor prognosis of HNSC. (A) TCGA and GTEx databases were combined to analyze the differential expression of NUDT21 in HNSC. (B) Survival analysis of APA factors in HNSC. (C) mRNA levels of NUDT21 across different stages of HNSC. (D) Correlation analysis between NUDT21 and PTEN, TP53, and RB1 in HNSC. (E) Representative Immunohistochemistry (IHC) Staining. NUDT21 protein expression was assessed in 25 paired clinical samples of OSCC and their corresponding NAT. The image shows representative IHC staining patterns observed in OSCC tissue. The scale bar: 100 nm. (F) Histoscore of NUDT21 in OSCC and NAT. Statistical significance was determined using a paired *t*‐test.

To investigate the clinical relevance of APA regulators in head and HNSC, we first interrogated the *NUDT21* expression profile within the Cancer Genome Atlas (TCGA) and Genotype‐Tissue Expression (GETx) database HNSC cohort. Our analysis revealed that *NUDT21* mRNA expression was significantly upregulated in tumor tissues (*n* = 519) compared to adjacent normal tissues (*n* = 44) (Figure 1A). This finding suggests a potential oncogenic role for NUDT21, aligning with emerging reports of its dysregulation in other malignancies [16, 17, 18, 19]. We next assessed the prognostic significance of NUDT21. Kaplan–Meier analysis revealed that patients with high NUDT21 expression exhibited significantly worse overall survival (Log‐rank *p* = 0.013; HR = 1.4) (Figure 1B). In contrast, CSTF2 levels showed no prognostic value (Log‐rank *p* = 0.55), whereas elevated PCF11 expression was unexpectedly associated with improved survival (Log‐rank *p* = 0.0025).

We next evaluated the relationship between NUDT21 and tumor progression. NUDT21 expression increased significantly with advancing clinical stage (Stage I–IV, *p* = 0.0261) (Figure 1C), whereas other APA core factors displayed no stage‐dependent trends (Figure S1). This comparative analysis highlights *NUDT21* as a key, prognostically‐relevant, pro‐tumorigenic APA factor in HNSC, justifying its selection as a high‐priority therapeutic target.

Given NUDT21's canonical function in promoting distal PAS usage [15], which often incorporates repressive elements like miRNA binding sites, we hypothesized that its upregulation might suppress the functional output of tumor suppressor genes. We explored this by correlating *NUDT21* mRNA levels (TPM) with those of key tumor suppressors. Interestingly, we observed significant *positive* correlations at the mRNA level between *NUDT21* and *PTEN* (R = 0.35, *p* = 0), *RB1* (R = 0.29, *p* = 4.4e‐12), and *TP53* (R = 0.11, *p* = 0.011) (Figure 1D). This initially counterintuitive positive correlation at the mRNA level masks a critical post‐transcriptional mechanism. We posit that this finding does not contradict the tumor‐suppressive role of *PTEN*, but rather exposes a classic hallmark of its dysregulation in cancer [20, 21]. High *NUDT21* activity likely forces *PTEN* transcripts into their long‐3'UTR isoforms. While these transcripts are stable enough to be detected at high levels (hence the positive TPM correlation), their long 3'UTRs render them highly susceptible to translational repression (e.g., via miRNA‐mediated inhibition) [22, 23]. This creates a scenario—classic in cancer—where high mRNA levels coexist with low functional protein output, thus driving the poor prognosis observed. This apparent paradox at the transcript level strongly necessitates a direct investigation into post‐transcriptional APA regulation and functional protein expression, which forms the central basis of our study. We posit that the high *NUDT21* gene promotes the production of long 3'UTR and low‐functioning proteins by tumor suppressor genes, thereby hindering their inhibitory effect on tumors. To further validate this hypothesis, we comprehensively analyzed multiple public RNA‐seq datasets from the Gene Expression Omnibus (GEO) database. These patient‐derived cohorts encompass a wide spectrum of clinical scenarios, including paired OSCC and normal adjacent tissues (NAT), the progressive stages from normal mucosa to pre‐malignant oral submucous fibrosis (OSF) and frank OSCC. By visualizing the transcriptomic read coverage across the *PTEN* locus using the Integrative Genomics Viewer (IGV), we observed a remarkably consistent pattern. Compared to normal or adjacent non‐tumor tissues, the read density extending into the distal 3'UTR region of *PTEN* was universally increased during malignant transformation and disease progression across analyzed datasets (Figure S2). This result strongly suggests that therapeutically targeting *NUDT21* would “reprogram” these transcripts to short‐3'UTR isoforms, thereby restoring their protein translation and tumor‐suppressive function.

Consistent with this post‐transcriptional repression model, we next examined NUDT21 protein expression in clinical OSCC tissues using a human oral cancer tissue microarray. Strikingly, immunohistochemical staining revealed that NUDT21 protein levels were markedly elevated in OSCC lesions compared with their corresponding NAT (Figure 1E). To quantify this difference, we calculated H‐scores for each paired sample. The results showed a significant increase in NUDT21 staining intensity in tumor tissues, further supporting its pathological upregulation in OSCC (Figure 1F). This protein‐level elevation is fully consistent with our APA‐based model: high NUDT21 expression in tumors would be expected to drive widespread long‐3'UTR usage in key tumor suppressors, thereby uncoupling transcript abundance from protein output. Thus, the tissue‐level IHC evidence (Figure 1E,F) provides an essential biological validation of NUDT21 overexpression in OSCC and reinforces its potential role as a post‐transcriptional regulator that functionally suppresses tumor‐inhibitory pathways.

### 2.2 Design, Synthesis, and Physicochemical Characterization of Nano‐APA‐Editors

**FIGURE 2:**
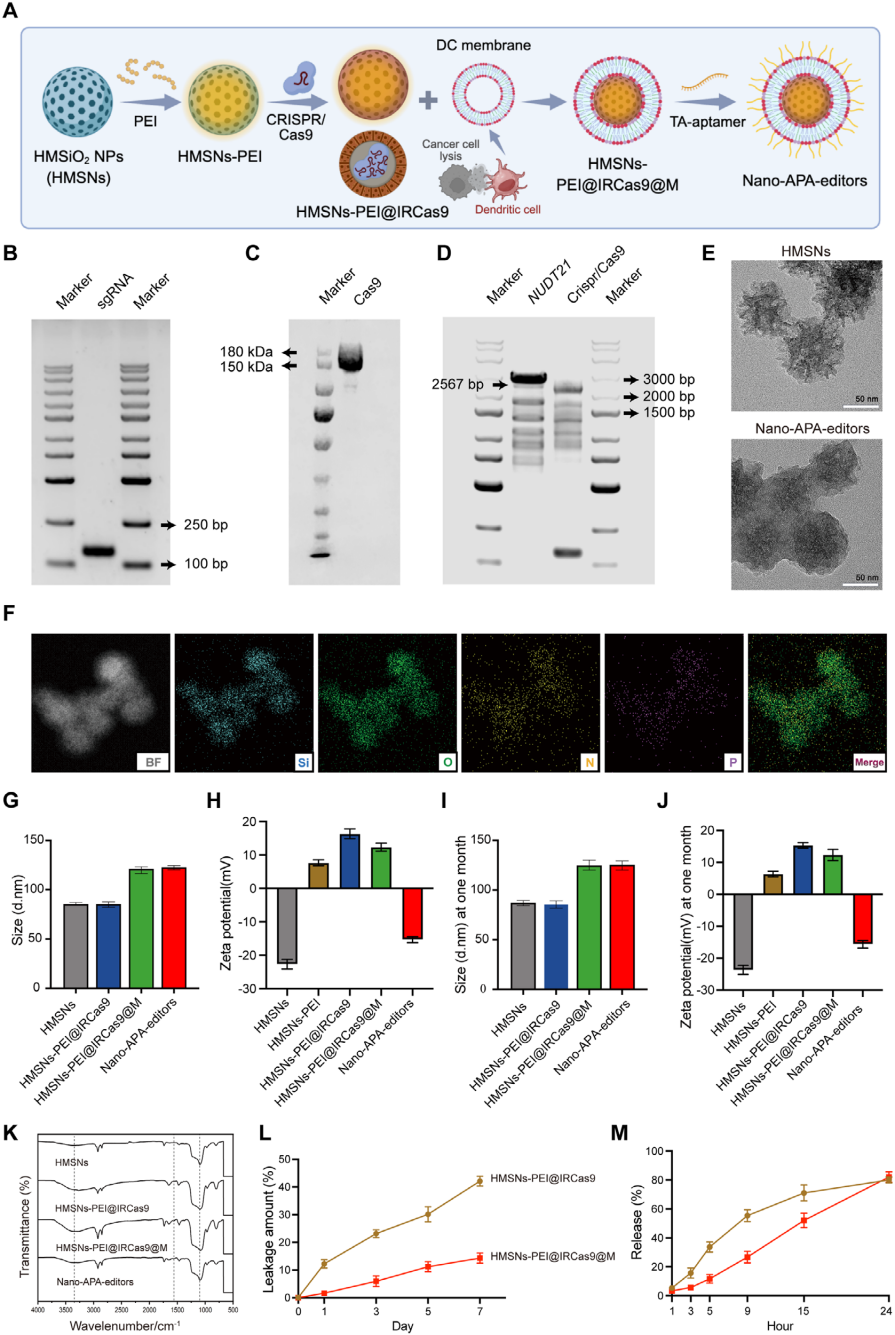
Preparation and characterization of Nano‐APA‐editors. (A) Synthesis pattern diagram of Nano‐APA‐editors. (B) The electrophoretic bands of sgRNA, (C) Cas9 protein, and (D) NUDT21 amplification fragment were detected by agarose gel electrophoresis. (E) Morphology assessment of HMSNs and Nano‐APA‐editors using transmission electron microscopy (TEM). (F) Visualization of elemental composition by EDS shows the BF image alongside separate mapping of silicon (blue), oxygen (green), nitrogen (yellow), and phosphorus (pink). (G) Size distribution and (H) zeta potential of HMSNs, HMSNs‐PEI, HMSNs‐PEI@IRCas9, HMSNs‐PEI@IRCas9@M, and Nano‐APA‐editors. (I) Size distribution and (J) zeta potential measured after 1 month at 4°C. (K) The FTIR analysis of HMSNs, HMSNs‐PEI@IRCas9, HMSNs‐PEI@IRCas9@M, and Nano‐APA‐editors. (L) RNA leakage curve of HMSNs‐PEI@IRCas9 and HMSNs‐PEI@IRCas9@M. (M) The releasing profiles of RNA in simulated body fluids (SBF) (pH 7.4) at 37°C.

Based on our therapeutic hypothesis, we rationally designed and constructed a multifunctional biomimetic nanoplatform, designated “Nano‐APA‐editors,” for the targeted in vivo delivery of CRISPR/Cas9 (IRCas9 for short, with sgRNA against *NUDT21*). The synthetic strategy is depicted in Figure 2A. The platform utilizes hollow mesoporous silica nanoparticles (HMSNs) as the inorganic core, chosen for their high specific surface area and excellent biocompatibility [24]. First, we successfully synthesized HMSNs using the improved Stöber method (Figure S3). These HMSNs were functionalized with PEI, imparting a positive charge, to form HMSNs‐PEI, which could then efficiently load the anionic sgRNA via electrostatic interaction [25]. The sgRNA is connected to Cas9 through non‐covalent interactions, resulting in the formation of HMSNs‐PEI@IRCas9. To bestow biomimetic properties, these nanoparticles were camouflaged with dendritic cell (DC) membranes that had been pre‐stimulated with cancer cell lysates, thereby inheriting tumor‐homing and immune‐evasive functionalities [26]. As a final step, a TA‐aptamer, known for its high affinity to HNSC cells [27], was conjugated to the membrane surface to achieve active, dual‐targeting specificity.

The successful preparation of the therapeutic payload components was confirmed by agarose gel electrophoresis. The bands corresponding to the sgRNA (approx. 100 bp), Cas9 protein (150–180 kDa), and the target *NUDT21* amplicon (2567 bp) were all validated (Figure 2B–D). Transmission electron microscopy (TEM) revealed the morphological changes during assembly. The pristine HMSNs appeared as uniform, spherical nanoparticles with a clear, porous structure. In striking contrast, the final Nano‐APA‐editors exhibited a distinct core–shell structure, where the dense HMSN core was visibly enveloped by a lighter‐contrast “corona,” characteristic of a cell membrane coating (Figure 2E). This composite structure was further confirmed by energy‐dispersive X‐ray spectroscopy (EDS) mapping, which showed the co‐localization of Silicon (Si) and Oxygen (O) from the core, along with Nitrogen (N) (from sgRNA/Cas9) and Phosphorus (P) (from the sgRNA phosphate backbone), confirming the successful loading of the payload (Figure 2F).

The stepwise layer‐by‐layer assembly was rigorously tracked using dynamic light scattering (DLS) and zeta potential measurements. The hydrodynamic diameter progressively increased from the original HMSNs (∼90 nm) to the membrane‐coated HMSNs‐PEI@IRCas9@M (∼120 nm), confirming the successful encapsulation of the cell membrane (Figure 2G). Among them, the modifications of PEI and the aptamer did not significantly affect the diameter of the nanoparticles. More definitive evidence was provided by the zeta potential analysis (Figure 2H). The bare HMSNs exhibited a negative charge (approx. ‐22 mV), which inverted to positive (approx. +8 mV) after PEI functionalization. After loading IRCas9, the potential further increased (approx. 17 mV). Critically, the potential decreases after the cell membrane is wrapped (approx. 11 mV), and further decreases after the aptamer is modified (approx. ‐16 mV). This characteristic charge‐reversal pattern not only confirms the successful membrane cloaking but also indicates a surface property that may aid in immune evasion and prolonged circulation [28, 29]. Second, the nanoconstructs demonstrated excellent colloidal stability, with no significant changes in size or zeta potential observed after 1 month of storage (Figure 2I,J). This assembly was also verified by Fourier‐transform infrared (FTIR) spectroscopy, which showed the sequential appearance of characteristic peaks from PEI, the IRCas9 complex, and the cell membrane overlaid on the foundational Si‐O‐Si peaks of the HMSNs (Figure 2K). The CRISPR/Cas9 system suffers from low delivery efficiency and rapid degradation, posing significant barriers to its therapeutic application [30]. To address these challenges, we camouflaged HMSNs‐PEI@IRCas9 with pretreated dendritic cell membranes (DCM), which not only enhanced colloidal stability but also imparted immune‐derived targeting capability. After storage at 4°C for seven days, HMSNs‐PEI@IRCas9 exhibited an RNA leakage rate of approximately 40%, whereas membrane coating reduced this value to only 13% (Figure 2L). Notably, under simulated physiological conditions, HMSNs‐PEI@IRCas9@M displayed a sustained and controlled release profile within 24 h (Figure 2M), effectively mitigating premature degradation and preserving its functional integrity in complex biological environments. Collectively, these data provide comprehensive evidence for the successful and robust fabrication of the Nano‐APA‐editors.

The primary goal of this study was to engineer a robust delivery vehicle to enhance the targeted in vivo application of CRISPR therapeutics, relying on the highly significant, system‐exclusive knockdown of NUDT21 (via RNA‐seq) as functional proof of efficacy. However, a limitation of the current study is the absence of direct genomic indel deep sequencing and unbiased genome‐wide off‐target profiling. Moving forward, as we adapt this nanoplatform for multi‐target or multi‐dimensional APA editors, deep sequencing will be essential to precisely map the genomic editing landscape prior to clinical translation.

### 2.3 Biomimetic Nano‐APA‐Editors Exhibit Enhanced Tumor‐Targeting and Potent Gene Silencing In Vitro

**FIGURE 3:**
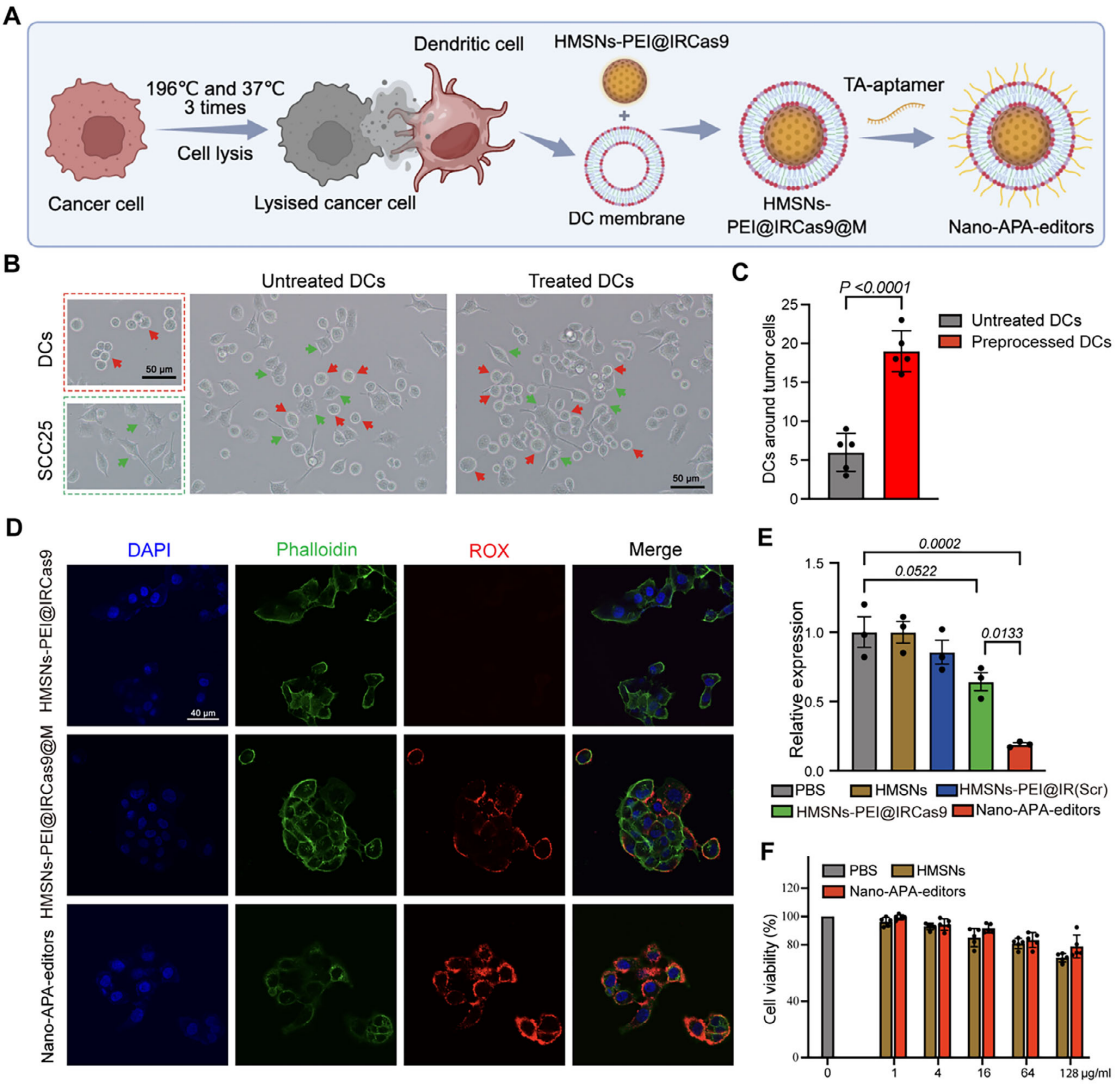
The powerful targeting ability and gene silencing effect of Nano‐APA‐editors. (A) Schematic diagram illustrating the dual‐targeting design of Nano‐APA‐editors. (B) Representative Images showing untreated DCs or treated DCs interacting with tumor cells at 200× magnification, and (C) Quantification of DC numbers around the tumor cells is given; *n* = 5 independent biological replicates. (D) Representative confocal fluorescence microscopy images showing the cellular uptake of HMSNs‐PEI@IRCas9, HMSNs‐PEI@IRCas9@M or Nano‐APA‐editors by SCC25 cells. The cytoskeleton was stained with phalloidin (green), and nuclei were stained with DAPI (blue). The ROX‐labeled IRCas9 is shown in red. Scale bars = 40 µm. (E) qPCR analysis was used to determine the levels of NUDT21 incubated with PBS, HMSNs, HMSNs‐PEI@IR(Scr), HMSNs‐PEI@IRCas9 or Nano‐APA‐editors. Data represent *n* = 3 independent biological replicates. (F) The cellular viability was measured as the dose increased; *n* = 3 independent biological experiments, with five technical replicates per experiment. All quantitative data are presented as the mean ± SD from at least three independent biological experiments. Statistical significance was determined using a one‐way analysis of variance (ANOVA) followed by Tukey's multiple comparisons test.

A cornerstone of our design is the biomimetic camouflaging strategy, which leverages a cancer‐“educated” DC membrane to confer homotypic targeting and immune‐evasive properties (Figure 3A). To validate this concept, we first generated “preprocessed DCs” by co‐culturing them with lysates from tumor cells (using SCC25 as an example), a process intended to “imprint” the DCs with cancer‐specific antigens and adhesion properties [31]. We then tested their affinity for SCC25 cells in a co‐culture assay. As visualized by microscopy, the preprocessed DCs (Treated DCs) demonstrated a marked tendency to recognize and aggregate around the target SCC25 cells, an interaction that was scarce in the “Untreated DCs” control group (Figure 3B). A quantitative analysis of this cell–cell interaction confirmed this observation, revealing a highly significant (*p < 0.0001*) increase in adhesion for the preprocessed DCs (Figure 3C). This result strongly validates our rationale that the membranes harvested from these “educated” DCs are enriched with factors that grant a high intrinsic affinity for the parent cancer cells.

We next investigated whether this enhanced targeting capability was successfully transferred to our nanoplatform and if it translated to superior cellular uptake. ROX‐labeled IRCas9 was incubated with SCC25 cells for 4 h. Confocal microscopy revealed that the non‐targeted HMSNs‐PEI@IRCas9 particles (lacking both membrane and aptamer) exhibited negligible cellular uptake. In striking contrast, nanoparticles cloaked with the educated DC membrane (HMSNs‐PEI@IRCas9@M) and the final dual‐targeting Nano‐APA‐editors (membrane plus aptamer) both showed massive intracellular accumulation, with a bright red ROX signal clearly co‐localizing within the phalloidin‐stained cytoplasm (Figure 3D). This highlights the dominant role of the ‘educated’ DC membrane in facilitating efficient endocytosis. Notably, the cellular uptake of Nano‐APA‐editors was markedly enhanced, which can be attributed to the high expression of AHNAK protein in OSCC cell lines (Figure S4). The TA aptamer specifically binds to AHNAK, thereby further improving the targeting capability. The TA‐aptamer is anticipated to play a critical, synergistic role in the complex in vivo milieu by enhancing the ‘first‐pass’ tumor recognition and subsequent retention within the tumor microenvironment, navigating challenges such as high interstitial fluid pressure and heterogeneous cell populations that are not recapitulated in vitro [32, 33].

Finally, we assessed the functional consequence of this enhanced delivery by quantifying *NUDT21* mRNA knockdown via qRT‐PCR. For the requisite negative control, an equivalent ribonucleoprotein complex was formed using Cas9 protein and a non‐targeting scramble sgRNA (sgScr) [34]. This control complex was loaded into the nanoplatform using identical procedures, hereafter referred to as HMSNs‐PEI@IR(Scr) for brevity. Treatment with control formulations (PBS, HMSNs, HMSNs‐PEI@IR(Scr)) resulted in no change to *NUDT21* expression. The non‐targeted HMSNs‐PEI@IRCas9 particles, which failed to enter cells efficiently, induced only a modest and non‐significant reduction in *NUDT21* mRNA (*p = 0.0522*). In sharp contrast, the Nano‐APA‐editors, leveraging their dual‐targeting design for superior cellular uptake, achieved a profound and statistically significant knockdown of *NUDT21* expression (*p = 0.0002* vs control) (Figure 3E). Meanwhile, the cell viability assay also demonstrates the superior safety profile of the nano‐formulation (Figure 3F). This demonstrates that our biomimetic and dual‐targeting strategy is essential for functionally delivering the IRCas9 payload and achieving robust silencing of the therapeutic target.

### 2.4 Nano‐APA‐Editors Potently Inhibit the Malignant Phenotypes of OSCC Cells In Vitro

**FIGURE 4:**
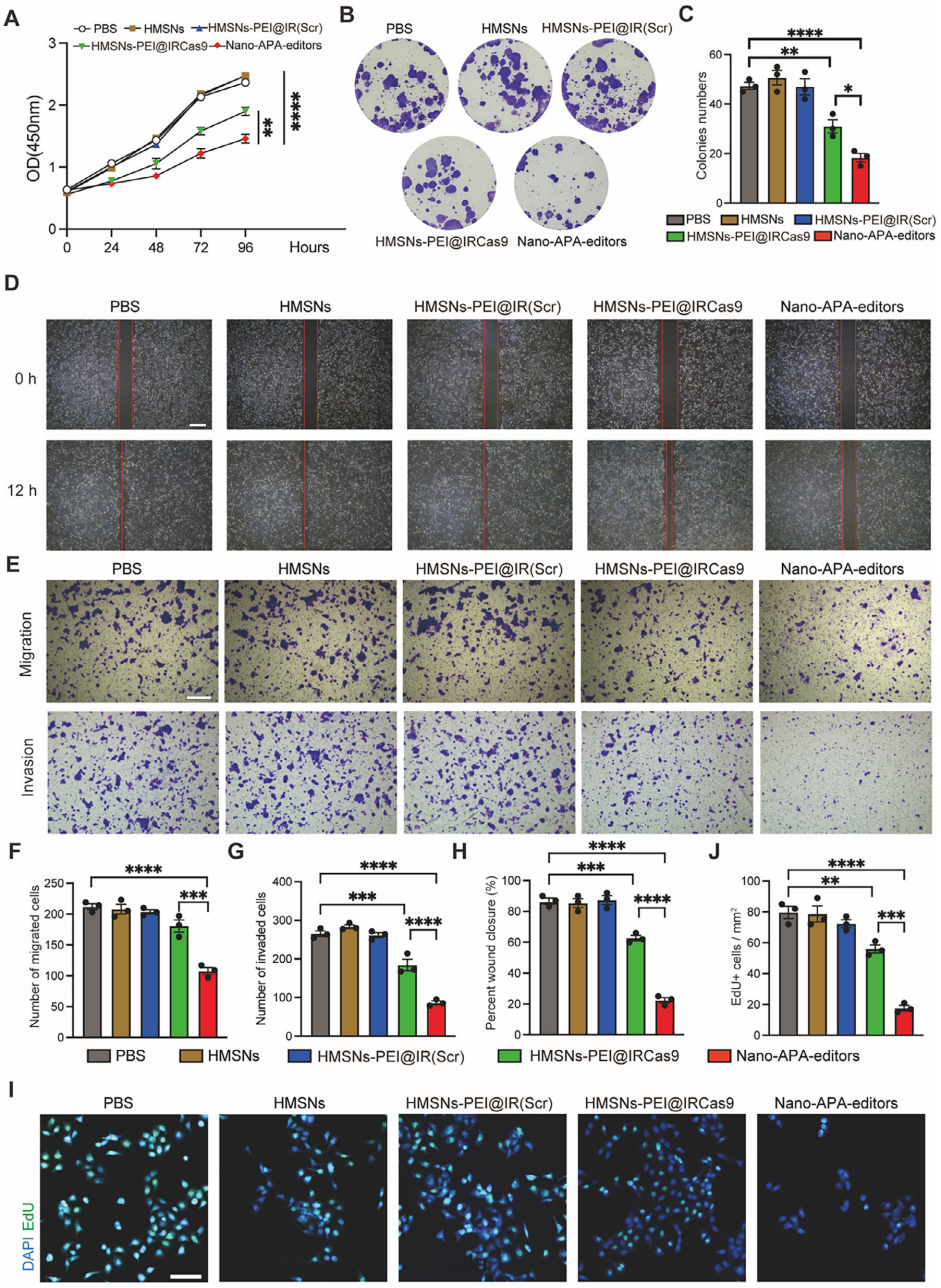
Antitumor activity of Nano‐APA‐editors. (A) Cell viability of SCC25 cells following treatments with PBS, HMSNs, HMSNs‐PEI@IR(Scr), HMSNs‐PEI@IRCas9, or Nano‐APA‐editors was quantified via the CCK‐8 assay; the detection wavelength is 450 OD. (B) Colony formation of SCC25 cells stained with crystal violet. (C) Quantification of SCC25 colonies after different treatments. (D) Representative images of SCC25 cells in the wound healing assay under different treatments. (E) The migration and invasion ability of SCC25 cells after different treatments was detected by a transwell assay, and the cells were stained with crystal violet. (F) The number of migrated and (G) invaded cells. (H) Statistical analysis of the wound closure percentage. I) Representative images of SCC25 cells stained by EdU and J) quantification of EdU‐positive cells. All quantitative data are presented as the mean ± SD. *n* = 3 independent biological experiments. Statistical significance was determined using a one‐way analysis of variance (ANOVA) followed by Tukey's multiple comparisons test. Asterisks indicate statistical significance: ^*^ *p* < 0.05, ^**^ *p* < 0.01, ^***^ *p* < 0.001, and ^****^ *p* < 0.0001.

Following the confirmation of efficient cellular uptake and target knockdown, we systematically evaluated the therapeutic efficacy of the Nano‐APA‐editors against the malignant behaviors of SCC25 cells in vitro. First, cell proliferation was monitored by a CCK‐8 assay. While the control groups (PBS, HMSNs, HMSNs‐PEI@IR(Scr)) all exhibited robust, overlapping growth curves, the Nano‐APA‐editors induced a profound and time‐dependent inhibition of cell viability, becoming highly significant at 72 and 96 h (*p* < 0.0001) (Figure 4A). Notably, the non‐targeted HMSNs‐PEI@IRCas9 group showed only a minor inhibitory effect, underscoring the critical importance of the biomimetic, dual‐targeting strategy for achieving therapeutic intracellular cargo delivery. The inertness of the HMSNs‐PEI@IR(Scr) group also confirms the excellent biocompatibility of the nanocarrier itself. This anti‐proliferative effect was further validated by a long‐term colony formation assay, where treatment with Nano‐APA‐editors resulted in a dramatic reduction in both the number and size of tumor cell colonies compared to all control groups (Figure 4B,C).

A hallmark of OSCC aggressiveness is its high potential for local invasion and distant metastasis [35]. We therefore investigated the impact of our nanoplatform on cell motility. In a wound‐healing assay, the control groups demonstrated rapid wound closure within 12 h, indicative of high migratory capacity. In stark contrast, the wound gap in the Nano‐APA‐editor‐treated cells remained largely open, translating to a significant inhibition of collective cell migration (Figure 4D,H). This anti‐migratory phenotype was corroborated by a Transwell migration assay, which showed a dramatic reduction in the number of cells that passed through the microporous membrane (Figure 4E,F upper panels). More critically, we assessed the cells' invasive potential using a Transwell assay with a Matrigel‐coated barrier, which mimics the basement membrane. The Nano‐APA‐editors almost completely abrogated the ability of SCC25 cells to invade through this extracellular matrix (Figure 4E,G lower panels). This potent inhibition of both migration and invasion—key steps in the metastatic cascade—suggests that *NUDT21* is a critical regulator of the cellular machinery responsible for OSCC motility. To elucidate the mechanism of this growth inhibition, we performed an EdU incorporation assay. The results clearly showed a significant decrease in the proportion of EdU‐positive (S‐phase) cells in the Nano‐APA‐editor treatment group (Figure 4I,J). This demonstrates that targeting *NUDT21* induces a potent cytostatic effect, primarily by arresting the cell cycle and preventing cells from entering or completing DNA synthesis. Taken together, these in vitro data comprehensively demonstrate that our biomimetic Nano‐APA‐editors, by effectively silencing *NUDT21*, can simultaneously suppress the core malignant phenotypes of OSCC: uncontrolled proliferation, migration, and invasion.

### 2.5 NUDT21 Silencing by Nano‐APA‐Editors Induces Global Transcriptomic Reprogramming of Oncogenic Pathways

**FIGURE 5:**
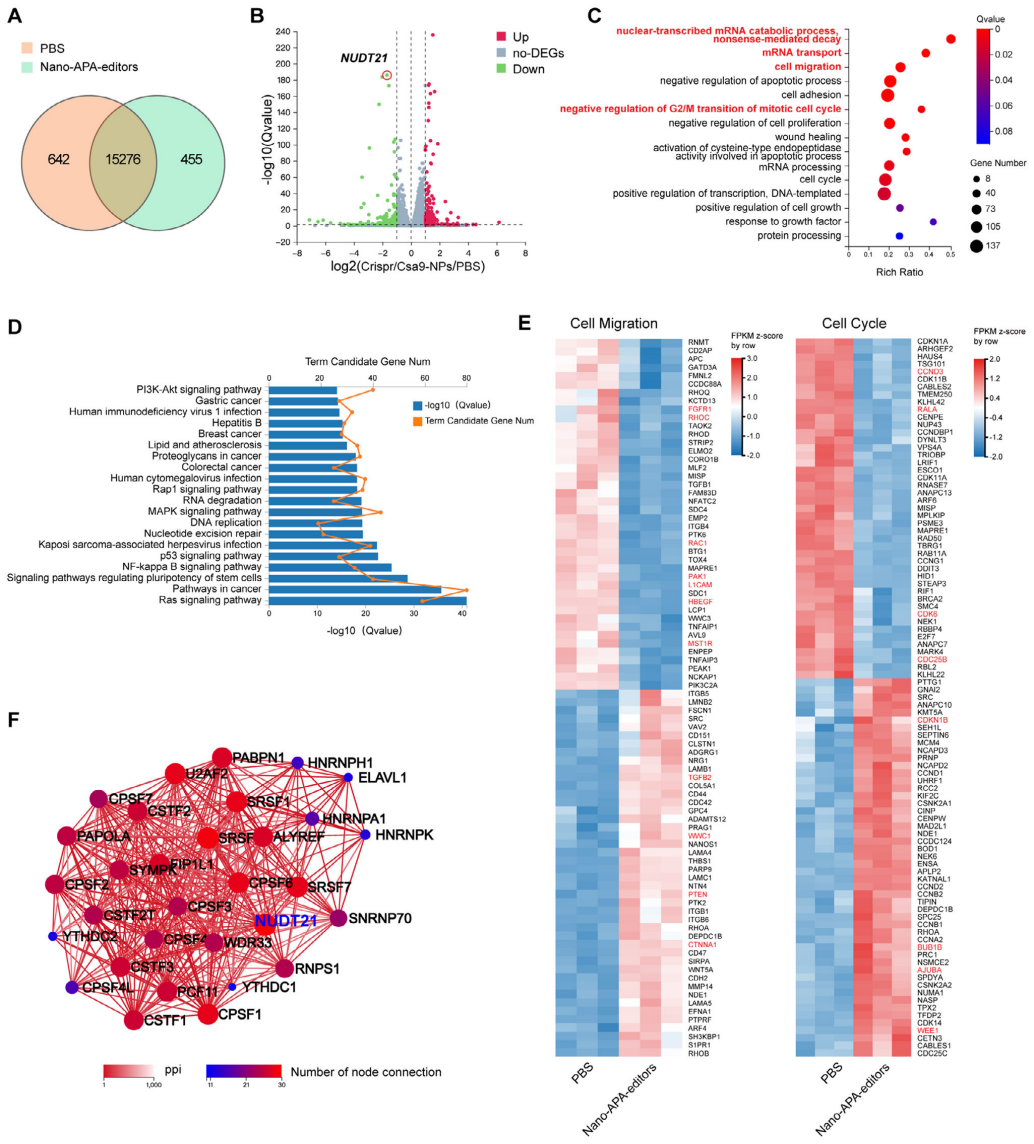
RNA‐seq analysis of SCC25 cells treated with PBS and Nano‐APA‐editors. (A) A Venn diagram revealed the number of genes transcribed in each treatment group. (B) Volcano plot of differentially expressed genes activated by Nano‐APA‐editors. (C) Gene Ontology‐biological process analysis of expressed genes (DEGs). (D) KEGG pathway analysis showed the signaling pathway. (E) Heatmap of genes which were associated with cell migration and cell cycle. (F) Gene interaction networks of NUDT21.

To elucidate the global molecular consequences of *NUDT21* knockdown, we performed comprehensive RNA‐sequencing (RNA‐seq) on SCC25 cells treated with either PBS or the Nano‐APA‐editors. The resulting transcriptomic profiles revealed a massive reprogramming of the cellular gene expression landscape. A total of 16373 genes were targeted, among which 455 genes were transcribed only in Nano‐APA‐editors–treated cells (Figure 5A). The volcano plot graphically illustrates this dramatic shift, highlighting a large number of differentially expressed genes (DEGs) between the two groups (Figure 5B). Critically, and serving as a primary validation of our platform's efficacy, *NUDT21* itself was identified as one of the most significantly downregulated transcripts (Figure 5B, red circle). This confirms that the Nano‐APA‐editors successfully engaged their intended target and induced potent gene silencing.

To understand the functional implications of this widespread transcriptomic change, we conducted Gene Ontology (GO) enrichment analysis on the DEGs. The results provided a striking molecular explanation for our in vitro phenotypic findings. Enriched biological processes were dominated by terms such as “negative regulation of cell proliferation,” “negative regulation of G2/M transition of mitotic cell cycle,” “cell migration,” “cell adhesion,” and “negative regulation of apoptotic process” (Figure 5C). Similarly, we also conducted GO enrichment analysis on the cellular components and molecular functions (Figure S5). This molecular signature aligns perfectly with the potent anti‐proliferative, cell‐cycle‐arresting, and anti‐migratory effects observed in Figure 4, confirming that *NUDT21* knockdown effectively shuts down the core programs that drive OSCC malignancy.

While this gene‐level analysis provides a crucial overview, NUDT21's function as an APA regulator suggests its primary impact may be at the transcript‐isoform level [36]. We therefore conducted a parallel analysis on differentially expressed transcripts (DETs). This revealed a distinct and complex landscape of transcript switching, as visualized by a volcano plot and heatmap of the top DETs (Figure S6). Notably, GO and KEGG enrichment analyses of these DETs not only reinforced the suppression of cell cycle and migration pathways, but also highlighted significant enrichment in terms related to “mRNA alternative polyadenylation', ”regulation of mRNA stability', “translational initiation', and ”post‐transcriptional protein modification' (Figures S7 and S8). This finding strongly supports our hypothesis that the observed phenotypes are a direct consequence of NUDT21‐mediated post‐transcriptional reprogramming, which precedes or occurs independently of simple gene‐level expression changes. This transcript‐centric view provides a more granular mechanistic link between NUDT21 silencing and the profound cellular reprogramming.

We next performed KEGG pathway analysis to map these DEGs onto established signaling cascades. This analysis revealed that the DEGs were significantly enriched in “Pathways in cancer,” the “p53 signaling pathway,” and the “PI3K‐Akt signaling pathway” (Figure 5D). The PI3K‐Akt pathway is a central, hyperactive oncogenic hub in HNSC, driving cell survival, proliferation, and therapeutic resistance [37, 38, 39]. Its identification as a primary target of NUDT21 silencing is a crucial mechanistic finding, strongly suggesting that the therapeutic effect of our Nano‐APA‐editors is mediated by the suppression of this master signaling cascade. This finding also provides a direct link to our initial hypothesis from Figure 1, as *PTEN* is the primary negative regulator of the PI3K‐Akt pathway [40, 41].

To identify the specific genetic drivers of these phenotypes, we examined the expression of key genes within the most enriched GO terms (Figure 5E). Within the “Cell Cycle” category, we observed a significant upregulation of the potent cell cycle inhibitor *CDKN1B* (p27), coupled with the marked downregulation of numerous pro‐proliferative cyclins such as *CCND3*, *CDK6*, and *CDC25B*. This “inhibitor‐up, promoters‐down” signature provides a precise molecular mechanism for the cell cycle arrest observed in Figure 4. Concurrently, within the “Cell Migration” category, we noted the downregulation of critical pro‐migratory factors, including the Rho‐family GTPases (*RHOC*) and integrins (*ITGB4*). The silencing of these genes, which are essential for cytoskeletal remodeling and focal adhesion, directly explains the profound inhibition of migration and invasion [42, 43, 44, 45]. Interestingly, genes such as *PTEN*, *TGFB2*, and *WWC1* were significantly upregulated. As a key APA regulatory factor, NUDT21 mediates the effects of Nano‐APA‐editors not only on the assembly of the APA complex but also on RNA processing and splicing (Figure 5F, Figures S7 and S8), thereby further promoting post‐transcriptional reprogramming. Collectively, these RNA‐seq data confirm that *NUDT21* knockdown rewires the entire oncogenic transcriptome, converging on the suppression of the PI3K‐Akt pathway and providing a clear molecular basis for the observed therapeutic effects.

It is important to note that this transcriptomic analysis provides a powerful, hypothesis‐generating snapshot of mRNA‐level reprogramming. While the observed upregulation of *CDKN1B* mRNA and downregulation of *RHOC* mRNA provide compelling molecular explanations for the in vitro phenotypes, we acknowledge that confirmation of these specific changes at the protein level was not the focus of this study. Instead, we prioritized the enrichment of the “PI3K‐Akt signaling pathway” as the most critical node, given its central role in OSCC [38, 39]. This led us to focus our subsequent mechanistic investigation squarely on its master upstream regulator, *PTEN*, and the specific APA mechanism governing its expression.

### 2.6 Nano‐APA‐Editors Remodel the 3'UTR Landscape to Promote Tumor Suppressor Isoform Switching

**FIGURE 6:**
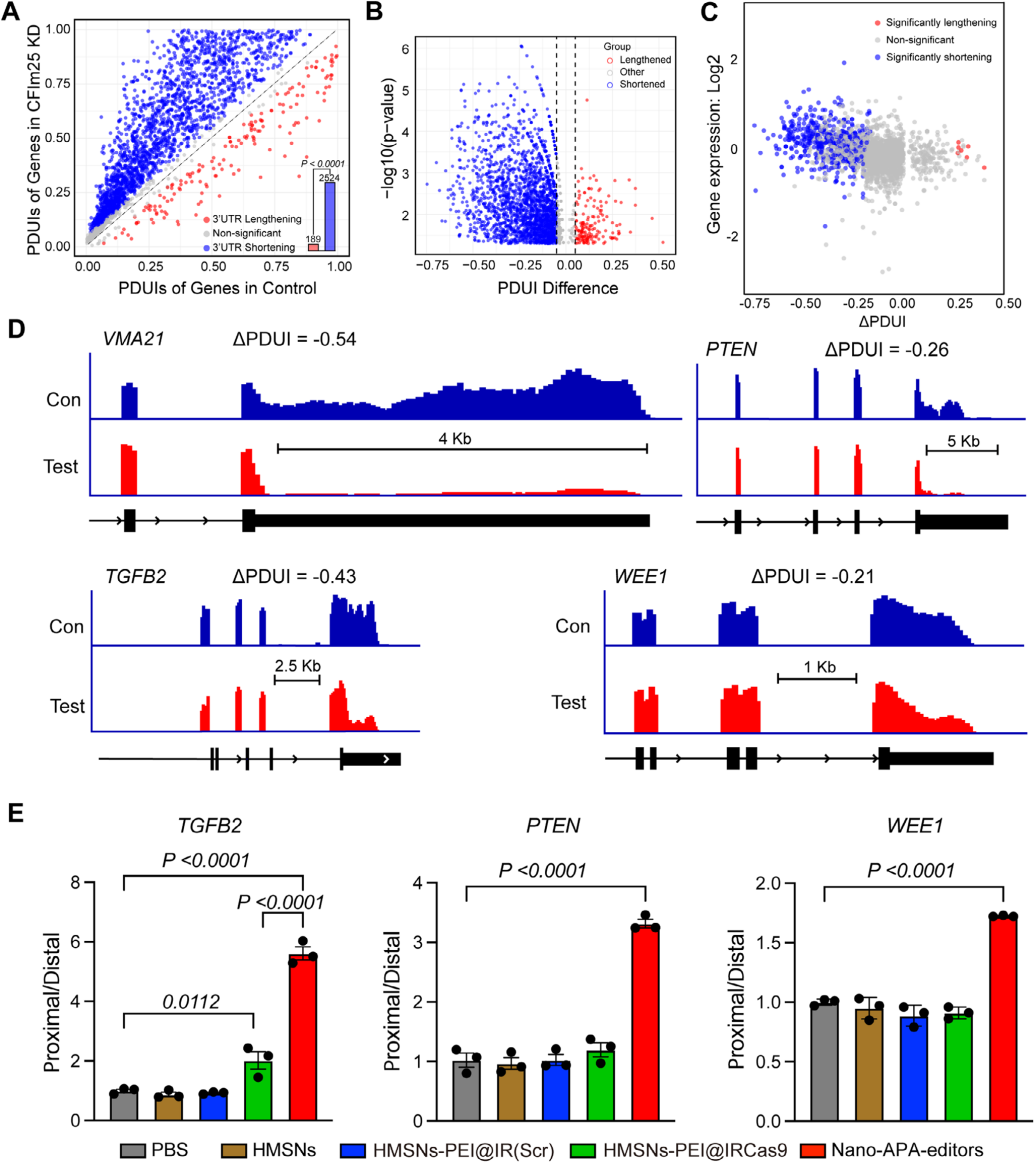
Nano‐APA‐editors remodel the 3'UTR landscape. (A) The differential usage of alternative 3'UTR isoforms was identified based on DaPars2, and a scatter plot was generated. After Nano‐APA‐editors treatment, mRNA exhibited significant shortening (*n* = 2524) or elongation (*n* = 189). (B) The volcano plot shows the mRNA that underwent elongation and shortening. (C) Correlation between distal PAS site usage and gene expression levels of control and Nano‐APA‐editors. (D) Representative RNA‐seq density plots along with ΔPDUI values for genes. (E) qPCR analysis to determine the levels of 3'UTR shortening. *n* = 3 independent biological replicates. Data are presented as mean ± SD. One‐way analysis of variance (ANOVA) with Tukey's multiple‐comparison test.

Our transcriptomic analysis in Figure 5 revealed a profound suppression of oncogenic pathways. We next sought to provide the definitive, underlying mechanism by which *NUDT21* silencing mediates this effect. Given that *NUDT21* is a core component of the CFIm complex that promotes dPAS usage [13, 14, 15, 46], we hypothesized that its knockdown would reprogram the global APA landscape. We performed 3'UTR‐focused RNA sequencing (Dapars2 analysis) on cells treated with Nano‐APA‐editors versus controls [47]. The results revealed a striking global shift in polyadenylation site preference. Surprisingly, a Percentage of Distal polyadenylation site Usage Index (PDUI) scatter plot showed that the vast majority of genes with altered 3'UTRs up the diagonal, indicating a transition from distal to proximal PAS usage, i.e., 3'UTR shortening. A total of 2,524 genes exhibited 3'UTR shortening, while only 189 genes showed 3'UTR lengthening. (Figure 6A). This global trend was further confirmed by a volcano plot, where the overwhelming majority of significantly altered transcripts (blue dots) exhibited a negative PDUI difference (Figure 6B). Pearson correlation = ‐0.22, it does reveal that transcripts with shorter 3'UTR generally exhibit. Genes with shortened 3'UTR generally showed a trend toward increased expression (Figure 6C). This also explains why the expression levels of genes that inhibit tumor cell proliferation, migration, and other functions are elevated.

We then focused on the specific tumor suppressor genes implicated in our earlier analyses. The genome browser tracks provide striking visual evidence of this APA switching (Figure 6D). Although the *VMA21* contains multiple polyadenylation sites, it typically utilizes only one of these sites across various cell types, tissues, and conditions, with a highly stable usage ratio. The PDUI value for the control group is close to zero, whereas after Nano‐APA‐editors treatment, the PDUI value is ‐0.54 (Figure 6D, top left). This not only confirms the reliability of our APA analysis workflow but also demonstrates that Nano‐APA induces significant alterations in PAS utilization. For the *PTEN* gene—the master regulator of the PI3K‐Akt pathway identified in Figure 5—control cells (Con) predominantly used a distal PAS, generating a long‐3'UTR transcript. Following treatment with Nano‐APA‐editors, the dPAS signal was markedly reduced, and a strong, new signal appeared at a proximal PAS, quantified by a △PDUI of ‐0.26 (Figure 6D, top right). This is a critical mechanistic link: the 3'UTR shortening of *PTEN* is known to liberate its mRNA from repression by multiple oncogenic miRNAs (e.g., miR‐21), thereby boosting its protein translation and enabling it to suppress the PI3K‐Akt pathway [48, 49, 50]. This same switching phenotype was observed for other key genes, including *TGFB2* (△PDUI = ‐0.43) and the G2/M checkpoint regulator *WEE1* (△PDUI = ‐0.21), providing a direct molecular explanation for the anti‐migratory and cell‐cycle‐arrest phenotypes observed in Figure 4 (Figure 6D bottom). Importantly, we also observed enrichment of these factors in GO analysis, and their correlation was consistent with that of *NUDT21*(Figure 6C). Nano‐APA‐editors induced extensive changes in the APA landscape. We also observed significant 3'UTR shortening of zinc finger proteins ZNF780A and ZNF33A (Figure S9), which are involved in key biological processes such as gene transcription regulation, cell proliferation, and development [51, 52]. Both proteins have transcriptional repressive functions, and their increased transcriptional activity may suppress the expression of certain tumor‐promoting genes, thereby inhibiting tumor progression [53, 54]. However, we found that the 3'UTR of TP53 underwent only moderate changes (△PDUI = ‐0.1), suggesting that Nano‐APA‐editors may affect the TP53 signaling pathway through alternative mechanisms.

To further validate this key finding, we designed a specific qRT‐PCR assay to quantify the ratio of Proximal‐to‐Distal (P/D) isoforms for these genes. The results were definitive (Figure 6E). For *TGFB2*, *PTEN*, and *WEE1*, the P/D ratio remained low and unchanged across all control groups (PBS, HMSNs, HMSNs‐PEI@IR(Scr)). The non‐targeted HMSNs‐PEI@IRCas9 group also failed to induce a shift, confirming that our biomimetic, dual‐targeting delivery system is essential for this effect. In sharp contrast, treatment with the Nano‐APA‐editors caused a dramatic and highly significant increase (*p* < 0.0001) in the P/D isoform ratio for all three genes (Figure 6E). This confirms that our Nano‐APA‐editors are not merely suppressing gene expression but are fundamentally “re‐engineering” the 3'UTRs of critical tumor suppressor transcripts, switching them from a translationally‐repressed (long) isoform to a translationally‐active (short) isoform. This APA‐reprogramming is the central molecular mechanism driving the potent anti‐tumor effects of our platform.

### 2.7 Nano‐APA‐Editors Demonstrate Superior Tumor Suppression and Excellent Biocompatibility In Vivo

**FIGURE 7:**
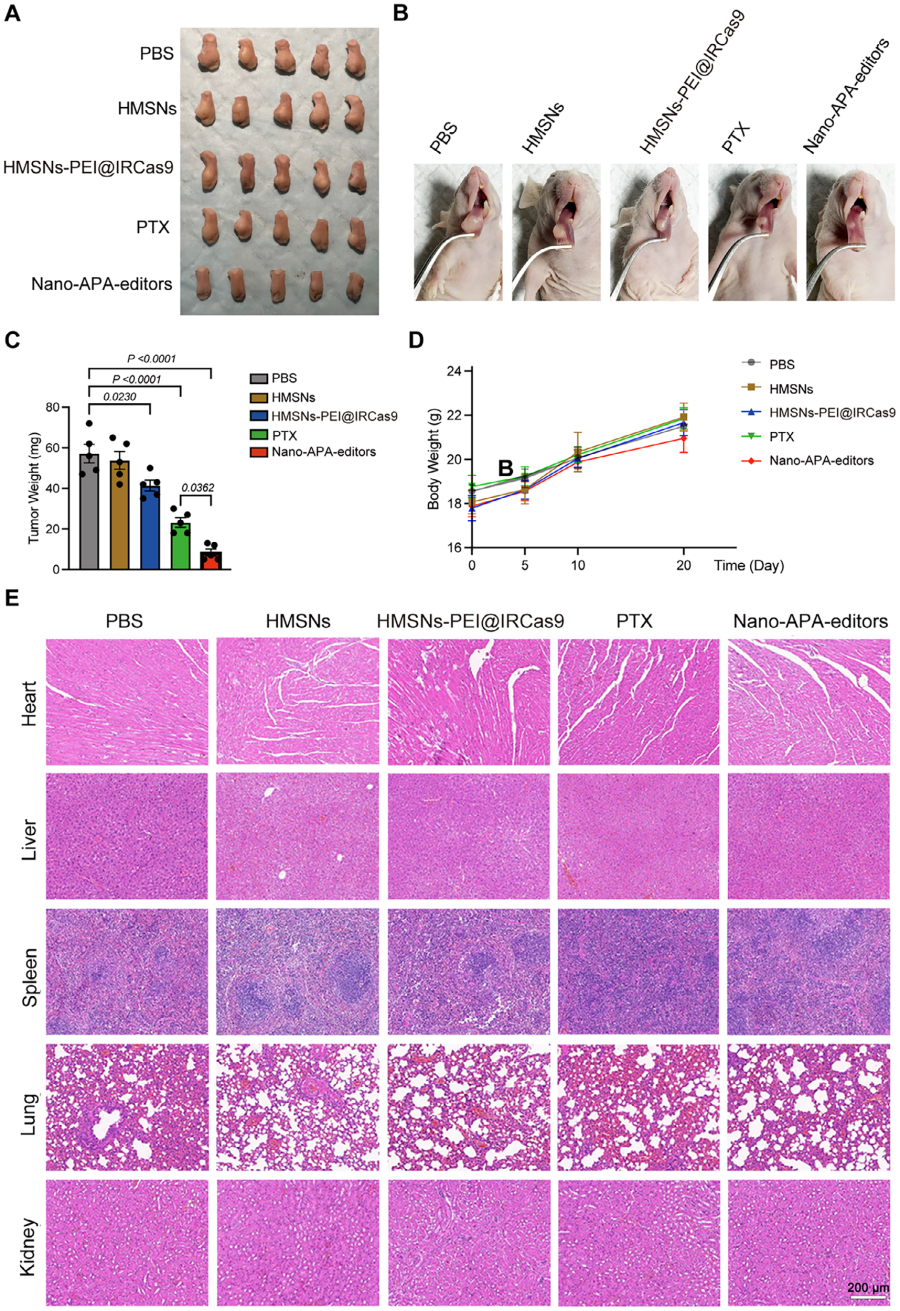
Nano‐APA‐editors demonstrate tumor suppression and biocompatibility. (A) Representative images of excised xenograft tumors and (B) the unexcised mouse tongue, with the tongue gently pulled out for imaging. (C) corresponding tumor weight extracted from BALB/c‐nu nude mice with PBS, HMSNs, HMSNs‐PEI@IRCas9, PTX or Nano‐APA‐editors treatment on day 20. (D) body weight changes. (E) H&E staining of the heart, liver, spleen, lung, and kidney. All quantitative data are presented as the mean ± SD. *n* = 5 mice per group. Statistical significance was determined using a one‐way analysis of variance (ANOVA) followed by Tukey's multiple comparisons test.

Encouraged by the potent in vitro anti‐tumor effects, we next assessed the therapeutic efficacy of our Nano‐APA‐editors in a clinically relevant orthotopic OSCC nude mouse model. After tumor establishment, mice were randomized into five groups and treated with PBS, HMSNs, HMSNs‐PEI@IRCas9, PTX (a first‐line chemotherapeutic) [55], or Nano‐APA‐editors. At the experiment's endpoint, the excised tumors visually demonstrated the potent therapeutic efficacy of our platform (Figure 7A). The control groups (PBS, HMSNs, and HMSNs‐PEI@IRCas9) all exhibited aggressive tumor growth, resulting in large tumor burdens (Figure 7B). This lack of efficacy in the HMSNs‐PEI@IRCas9 group confirms our in vitro finding that the biomimetic, dual‐targeting strategy is absolutely essential for functional delivery in vivo.

Quantitative analysis of the final tumor weights provided a striking confirmation of our platform's superiority (Figure 7C). While the standard chemotherapy (PTX) achieved a significant reduction in tumor weight compared to the PBS control (*p* < 0.0001), the Nano‐APA‐editors induced a far more profound tumor regression (*p* < 0.0001 vs PBS). Most importantly, the anti‐tumor effect of the Nano‐APA‐editors was significantly greater than that of the PTX group (*p* = 0.0362). This superior in vivo performance can be attributed to the “active” and “biomimetic” targeting design, which, as demonstrated in Figure 3, facilitates enhanced tumor accumulation and cellular uptake. This allows for site‐specific delivery of the APA‐reprogramming payload, a highly targeted mechanism that appears more effective than the non‐specific cytotoxicity of PTX.

A critical aspect of any nanomedicine is its systemic biocompatibility [56]. Hemolysis assay results showed that Nano‐APA‐editors did not induce significant hemolysis (Figure S10). We rigorously monitored the health of the mice throughout the treatment period. Reassuringly, no significant body weight loss was observed in any of the groups; in fact, all mice, including those treated with Nano‐APA‐editors, exhibited a steady increase in body weight, indicative of good general health and a lack of systemic toxicity (Figure 7D). To further investigate potential long‐term toxicity, we performed histological analysis on the major organs (heart, liver, spleen, lung, and kidney) at the end of the study. Hematoxylin and eosin (H&E) staining revealed no signs of pathological damage, inflammation, or lesions in any organ from any treatment group (Figure 7E). This comprehensive safety profile, demonstrating both a lack of systemic toxicity and no organ damage, is a testament to the excellent biocompatibility of the HMSN core and the immune‐evasive properties of the dendritic cell membrane camouflage. Collectively, these results demonstrate that the Nano‐APA‐editors are not only a potent anti‐tumor platform that outperforms the standard‐of‐care, but also a highly safe and biocompatible system for in vivo gene‐editing therapy.

A significant consideration in interpreting these in vivo results is the use of an immunodeficient (nude mouse) xenograft model [57]. While this model is the standard for unequivocally assessing the direct cytotoxic and cytostatic effects of a therapeutic agent on human‐derived cancer cells, it inherently precludes a full evaluation of the nanoplatform's complex interactions with a complete immune system [58]. The “immune‐evasive” properties conferred by the DC membrane camouflage are strongly supported by its superior efficacy over non‐camouflaged particles, likely by preventing clearance by the innate immune system (e.g., macrophages) still present in nude mice [59]. However, any potential immunomodulatory roles of the DC membrane, such as the presentation of cancer‐lysate antigens to T‐cells, could not be assessed. Future studies in syngeneic, immunocompetent models are warranted to explore these potentially synergistic immunotherapeutic dimensions.

### 2.8 Nano‐APA‐Editors Suppress Tumor Growth by Inducing Apoptosis and Validating the APA‐Reprogramming Axis In Vivo

**FIGURE 8:**
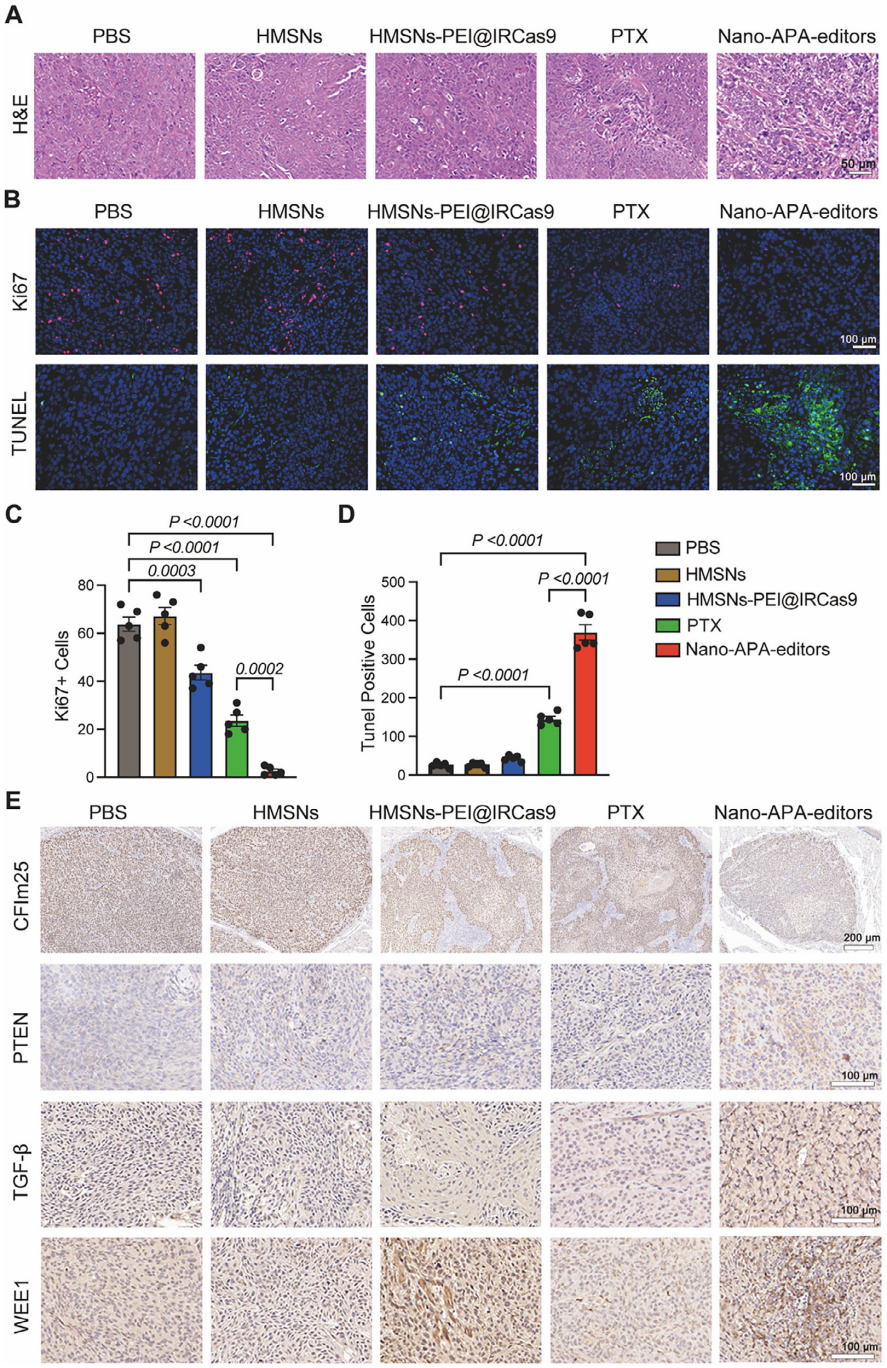
Nano‐APA‐editors induced apoptosis and validating the APA‐reprogramming axis. (A) H&E staining of the harvested tumors. (B) Representative image of Ki67 and TUNEL staining. (C) The number of Ki67 positive cells and (D) Tunel positive cells. (E) Immunohistochemistry analysis of CFIm25, PTEN, TGF‐β, and WEE1. All quantitative data are presented as the mean ± SD. *n* = 5 independent tumor samples. Statistical significance was determined using a one‐way analysis of variance (ANOVA) followed by Tukey's multiple comparisons test.

To elucidate the cellular and molecular mechanisms behind the potent tumor suppression observed in Figure 7, we conducted a thorough histological and immunohistochemical (IHC) analysis of the excised orthotopic tumors. H&E staining revealed that tumors from the control groups (PBS, HMSNs, HMSNs‐PEI@IRCas9) consisted of dense, viable, and highly proliferative cancer cells. The PTX‐treated group showed some reduction in tumor density, but the Nano‐APA‐editors‐treated group was strikingly different, displaying extensive areas of coagulation necrosis, nuclear shrinkage, and cellular disintegration, indicative of widespread and effective tumor cell killing (Figure 8A).

This potent therapeutic effect was further dissected using immunofluorescence staining for proliferation (Ki67) and apoptosis (TUNEL) (Figure 8B). As quantified in Figure 8C, control‐treated tumors exhibited a very high proliferation index (Ki67^+^ cells). While PTX treatment significantly reduced proliferation (*p =* 0.0003), the Nano‐APA‐editor treatment nearly eradicated the proliferative cell population, demonstrating a significantly superior anti‐proliferative effect (*p =* 0.0002 vs PTX). This result perfectly mirrors our in vitro EdU data (Figures 4I,J). Conversely, TUNEL staining, a marker for apoptotic cells, was negligible in all control groups. Both PTX and Nano‐APA‐editors significantly induced apoptosis (*p <* 0.0001), but the apoptotic signal in the Nano‐APA‐editors group was profoundly more intense and widespread, indicating a massive induction of programmed cell death (Figure 8B,D).

Finally, we performed IHC to provide the definitive in vivo validation of our proposed APA‐reprogramming mechanism (Figure 8E). This analysis provided a clear molecular snapshot that closes the loop on our entire study. First, we confirmed target engagement: tumors treated with Nano‐APA‐editors showed a dramatic reduction in the protein expression of our target, CFIm25 (*NUDT21*), compared to the strong staining observed in all control groups. This provides unequivocal evidence that our platform successfully delivered its sgRNA payload and silenced the target gene in vivo. Second, and most critically, we assessed the downstream consequence of this silencing. In exact accordance with our in vitro 3'UTR‐shortening hypothesis, the silencing of *NUDT21* led to a striking *upregulation* of PTEN protein expression. This demonstrates that by forcing the *PTEN* transcript into its shorter, translationally‐active isoform, our Nano‐APA‐editors successfully restored the protein levels of this master tumor suppressor. The same restorative upregulation was also observed for TGF‐β and WEE1 [60, 61]. This in vivo molecular data is the final piece of the puzzle, confirming that the Nano‐APA‐editors function by re‐engineering the 3'UTR landscape to restore the expression of key tumor suppressors. This NUDT21‐down/PTEN‐up axis provides powerful in vivo validation for our central hypothesis. While the upregulation of the lipid phosphatase PTEN is the canonical and most powerful brake on the PI3K‐Akt pathway [40, 41]—which our RNA‐seq identified as a key node —we acknowledge that we did not directly confirm the subsequent in vivo suppression of its downstream effectors, such as the phosphorylation of Akt (p‐Akt). Future studies quantifying p‐Akt and other pathway members would provide a more complete picture of the subsequent signaling cascade. Nonetheless, the successful restoration of PTEN protein expression in vivo is, in itself, a profound therapeutic achievement and the most critical validation of our APA‐reprogramming strategy.

It should be noted that NUDT21 functions as a global regulator of APA. Consequently, the tumor regression achieved by the nano‐editor is likely driven by the simultaneous transcriptomic remodeling of a broad network of tumor suppressors, potentially extending beyond the specific targets (e.g., *WEE1*, *TGF‐β*) highlighted in this study. Within this multi‐target paradigm, *PTEN* serves as a highly representative and biologically critical model gene to elucidate the APA‐mediated mechanism. Therefore, it reflects a wider, network‐level anti‐tumor response elicited by *NUDT21* silencing.

## 3 Conclusion

In summary, this study identifies the alternative polyadenylation regulator NUDT21 as a critical, prognostically significant, and high‐value therapeutic target in OSCC. Our findings establish a strong mechanistic link whereby NUDT21's pro‐tumorigenic drive is associated with its role in reprogramming the 3'UTR landscape. We show that this reprogramming facilitates the translational suppression of a broader network of tumor suppressors, with PTEN serving as a critical representative hallmark. To precisely exploit this post‐transcriptional vulnerability, we rationally engineered a sophisticated “Nano‐APA‐editor” platform. This nanoconstruct integrates biomimetic, cancer‐educated DC membrane camouflage with active aptamer‐based dual‐targeting, achieving high in vivo targeting specificity and efficacy. By successfully delivering its IRCas9 payload, this platform silences NUDT21, therapeutically “rewires” the pathogenic APA signature, and restores the functional protein expression of these key tumor suppressors. This mechanism culminates in profound tumor regression that surpasses conventional chemotherapy, all while maintaining an excellent systemic safety profile. This work not only provides a powerful, clinically‐translatable nanostrategy for OSCC but, more broadly, validates the targeted reprogramming of APA as a potent and entirely new therapeutic modality for cancer gene therapy.

## 4 Experimental Section

### 4.1 Synthesis of Hollow Mesoporous Silica Nanoparticles (HMSNs)

HMSNs were synthesized via a modified two‐step sol–gel process. First, 2 g of CTAB was dissolved in 30 mL of deionized (DI) water at 95°C under vigorous stirring, followed by the addition of 30 mg of TEA. After 1 h of equilibration, 1.5 mL of TEOS was added dropwise and stirred for another 1 h to form mesoporous silica nanoparticles (MSNs). To create the hollow structure, the as‐prepared MSNs were dispersed in 50 mL of 0.1 m sodium carbonate (Na_2_CO_3_) solution and heated at 60°C for 5 h to selectively etch the inner silica core. The obtained products were centrifuged (10 000 × g, 10 min) and washed twice with DI water and ethanol. To remove the CTAB template, the samples were extracted in 1 wt.% NaCl/methanol solution for 8 h at room‐temperature, followed by centrifugation and vacuum drying. The final HMSNs exhibited a well‐defined hollow cavity and uniform mesoporous shell, as confirmed by transmission electron microscopy (TEM) and nitrogen adsorption–desorption analysis.

### 4.2 Synthesis and Characterization of Nano‐APA‐Editors

The as‐prepared HMSNs were dispersed in 10 mL of DI water (1 mg mL^−^^1^) and sonicated for 10 min. Then, 10 mg of branched PEI (Mw ≈ 25 kDa) was added and stirred at room‐temperature for 6 h to allow electrostatic adsorption of PEI onto the negatively charged silica surface. The products were collected by centrifugation (10 000 × g, 10 min) and washed three times with DI water to remove excess PEI. The resulting HMSNs‐PEI exhibited a positive surface potential, facilitating nucleic acid loading. sgRNA and recombinant Cas9 protein were pre‐mixed at a molar ratio of 1:1.2 and incubated for 15 min at room‐temperature to allow complex formation via non‐covalent electrostatic and hydrogen‐bond interactions. Subsequently, HMSNs‐PEI (1 mg) was added into 1 mL of sgRNA‐Cas9 solution and incubated for 2 h at room‐temperature with gentle shaking. The obtained HMSNs‐PEI@IRCas9 nanocomplexes were purified by centrifugation (10 000 × g, 10 min) and washed twice with RNase‐free PBS to remove unbound complexes. Murine bone‐marrow‐derived dendritic cells (DC2.4) were cultured, and then, DC membranes were extracted and purified by using hypotonic centrifugation. The purified DC membranes were extruded through 400 and 200 nm polycarbonate membranes sequentially to obtain uniform vesicles. HMSNs‐PEI@IRCas9 and DC membrane vesicles (mass ratio = 1:1) were co‐extruded through 200 nm membranes using a mini‐extruder for ten cycles to form biomimetic nanoparticles (HMSNs‐PEI@IRCas9@M). The targeting aptamer (TA) was synthesized with a cholesterol modification at its 3′ end to enable spontaneous insertion into the lipid bilayer. HMSNs‐PEI@IRCas9@M (1 mg mL^−^^1^) were incubated with Chol‐TA (final concentration 2 µm) at 37°C for 30 min under gentle shaking. The hydrophobic cholesterol moiety facilitated the anchoring of TA onto the outer DC membrane through lipid‐bilayer insertion. The resulting Nano‐APA‐editors were collected by centrifugation (10 000 × g, 10 min) and washed with PBS to remove unbound aptamers.

### 4.3 Characterization

The morphology and hollow mesostructure of HMSNs, HMSNs‐PEI, and the final HMSNs‐PEI@IRCas9@M‐TA nanocomplex were examined by transmission electron microscopy (TEM, JEM‐F200, JEOL, Japan) operated at 200 kV. Elemental mapping images of the composite nanoparticles were obtained using a high‐resolution transmission electron microscope (Tecnai G2 F30, FEI, Holland) to confirm the homogeneous distribution of Si, N, P, and S elements. The hydrodynamic size distribution and zeta potential of nanoparticles at each modification step were measured using a Zetasizer Nano ZS90 (Malvern Instruments, UK). The crystal structure of HMSNs was analyzed by X‐ray diffraction (XRD, X'Pert PRO, PANalytical B.V., Netherlands) at room temperature. Fourier transform infrared (FT‐IR) spectra were recorded on a Thermo Scientific Nicolet iN10 spectrometer to identify characteristic functional groups after surface modification. The fluorescence colocalization and cellular uptake of IRCas9 were observed using confocal laser scanning microscopy (CLSM, Leica TCS SP8 and Zeiss Axio Zoom.V16).

### 4.4 Cell Cultures

The human oral squamous cell carcinoma cell line SCC25 was obtained from the China Center for Type Culture Collection (CCTCC, Wuhan, China; Cat. No. GDC0451). Cells were cultured in DMEM medium supplemented with 10% fetal bovine serum (FBS), 100 U/mL penicillin, and 100 µg/mL streptomycin. The cells were maintained at 37°C in a humidified incubator with 5% CO_2_ and routinely tested to ensure they were free of mycoplasma contamination. When the cell confluence reached approximately 80%, the cells were sub‐cultured using 0.25% trypsin–EDTA for further experiments.

### 4.5 Animals

The 8‐week‐old female BALB/c nude mice were purchased from WeiTong LiHua Co. Animals were maintained under specific pathogen‐free conditions. All animal experimental procedures were approved by the Animal Care and Use Committee of the Medical Research Institute, Wuhan University (ethical approval number S07923090C).

### 4.6 Human Tissue Samples and Immunohistochemistry (IHC)

Human tissue samples were obtained with approval from the Ethics Committee of the School and Hospital of Stomatology, Wuhan University (IRB‐ID: 2024A59). Tumor tissues and corresponding NAT were collected from patients diagnosed with OSCC. Tissue microarrays were constructed from formalin‐fixed, paraffin‐embedded specimens. Immunohistochemical staining was performed to evaluate NUDT21 expression according to standard protocols.

### 4.7 Statistical Analysis

All statistical analyses were performed using GraphPad Prism software (version 10.0). Comparisons between two groups were analyzed using an unpaired two‐tailed Student's *t* tests. For analyses involving three or more independent groups, one‐way ANOVA followed by Tukey's post hoc test was applied. Unless otherwise specified, all in vitro quantitative data are presented as the mean ± standard deviation (SD) from at least three independent biological replicates. A *p*‐value < 0.05 was considered statistically significant. Statistical significance in the figures is denoted as follows: ^*^ *p* < 0.05, ^**^ *p* < 0.01, ^***^ *p* < 0.001, and ^****^ *p* < 0.0001; ns indicates not significant.

## Author Contributions

**Yiran Ao**: methodology, visualization, writing – original draft, validation, formal analysis, conceptualization, software, data curation. **Bin Gu**: writing – original draft, conceptualization, methodology, software, data curation, validation, visualization, formal analysis. **Kun Tan**: methodology, data curation, validation, formal analysis. **Qin Zhao**: writing – review and editing, conceptualization, investigation, funding acquisition, validation, formal analysis, resources, project administration, supervision. **Hui Zhao**: methodology, validation. **Zhengjun Shang**: investigation, project administration, supervision, writing – review and editing, validation, resources.

## Funding

This work is supported by the National Natural Science Foundation of China (82571092) and the China Postdoctoral Science Foundation under Grant Number 2025M772606.

## Conflicts of Interest

The authors declare no conflicts of interest.

## Supporting information




**Supporting File**: advs76126‐sup‐0001‐SuppMat.docx.

## Data Availability

The data that support the findings of this study are available from the corresponding author upon reasonable request.
