# Supplementary material for: A Biomimetic Dual‐Targeting Nano‐APA‐Editor Reprograms the 3'UTR Landscape for Tongue Squamous Cell Carcinoma Therapy
Source: Adv Sci (Weinh). 2026 Jun 15:e76126. Online ahead of print. doi: 10.1002/advs.76126 (PMC13336823; doi:10.1002/advs.76126)
Supplement: Supplementary file 1 — Supporting File: advs76126‐sup‐0001‐SuppMat.docx. [file ADVS-9999-e76126-s001.docx]

Supporting Information

**A Biomimetic Dual-Targeting Nano-APA-Editor Reprograms the 3'UTR Landscape for Tongue Squamous Cell Carcinoma Therapy**

*Yiran Ao, Bin Gu, Hui Zhao, Kun Tan, Qin Zhao*, and Zhengjun Shang**

Dr. Yiran Ao, Bin Gu, Hui Zhao, Kun Tan, Prof. Qin Zhao, and Zhengjun Shang

State Key Laboratory of Oral & Maxillofacial Reconstruction and Regeneration, Key Laboratory of Oral Biomedicine Ministry of Education, Hubei Key Laboratory of Stomatology, School & Hospital of Stomatology, Wuhan University, Wuhan 430079, P. R. China

Prof. Zhengjun Shang

Taikang Center for Life and Medical Sciences, Wuhan University, Wuhan 430071, P. R. China

E-mail: shangzhengjun@whu.edu.cn (Zhengjun Shang); zhaoqin@whu.edu.cn (Qin Zhao)


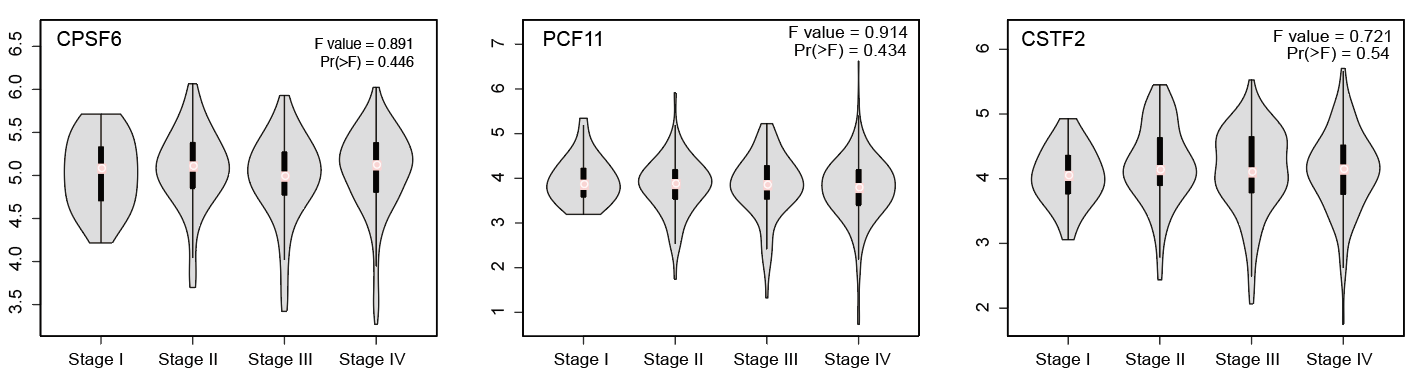


**Figure S1****. mRNA levels of other APA factors across different stages of HNSC.**

**Figure S2. The IGV tracks displaying the normalized RNA-seq read coverage of PTEN in the GEO datasets.**

A) Representative IGV tracks from dataset GSE255993 and B) GSE222673, comparing ANT and OSCC samples. C) IGV tracks from dataset GSE274203 illustrating the progressive PTEN 3'UTR lengthening from normal oral mucosa to OSF and OSCC.

**
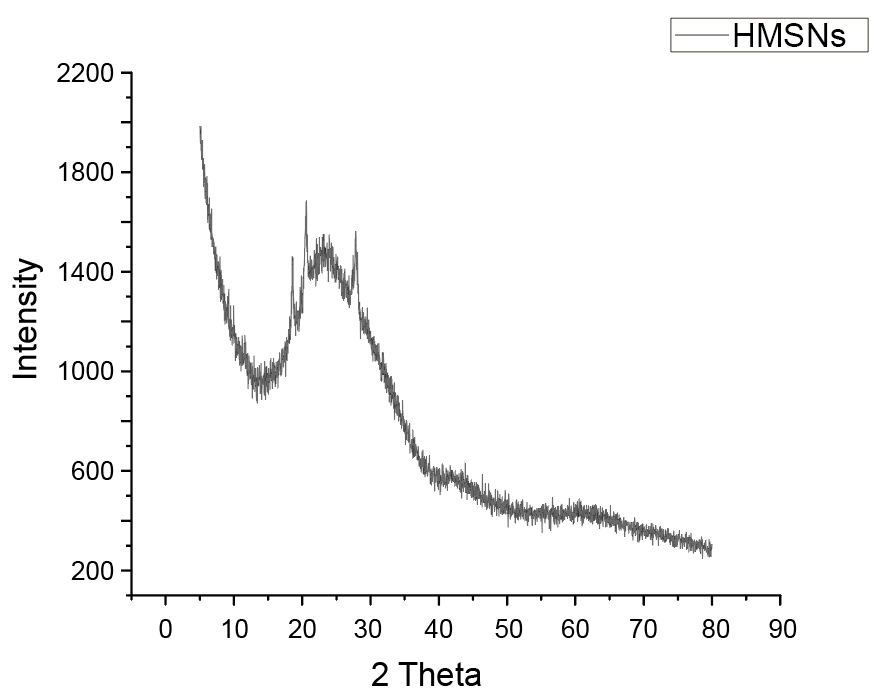
**

**Figure S3. The XRD patterns of HMSNs.**


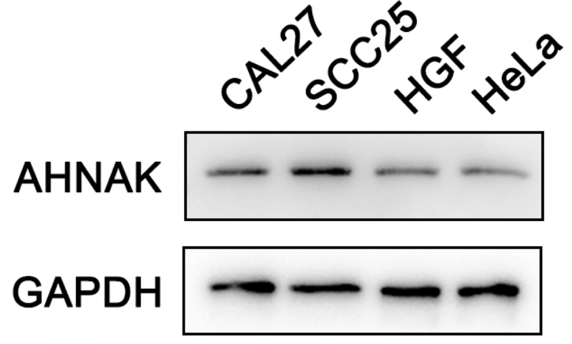


**Figure S4. Western blotting was used to detect the protein level of AHNAK in the cells.**


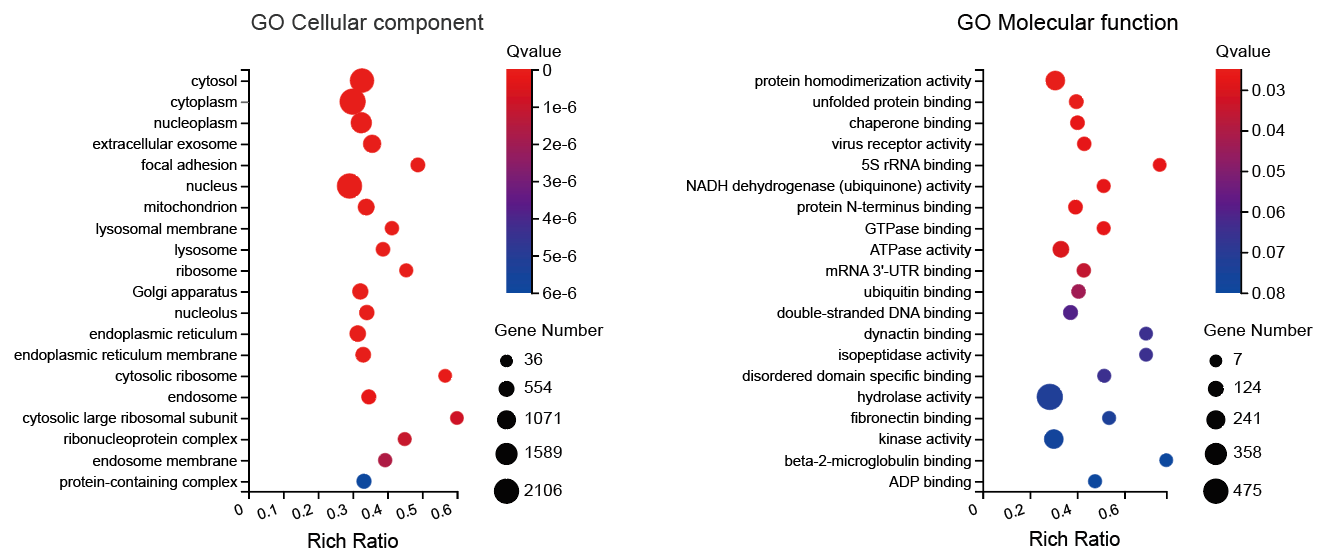


**Figure S5. Gene Ontology-cellular component molecular function and molecular function analysis of expressed genes (DEGs).**


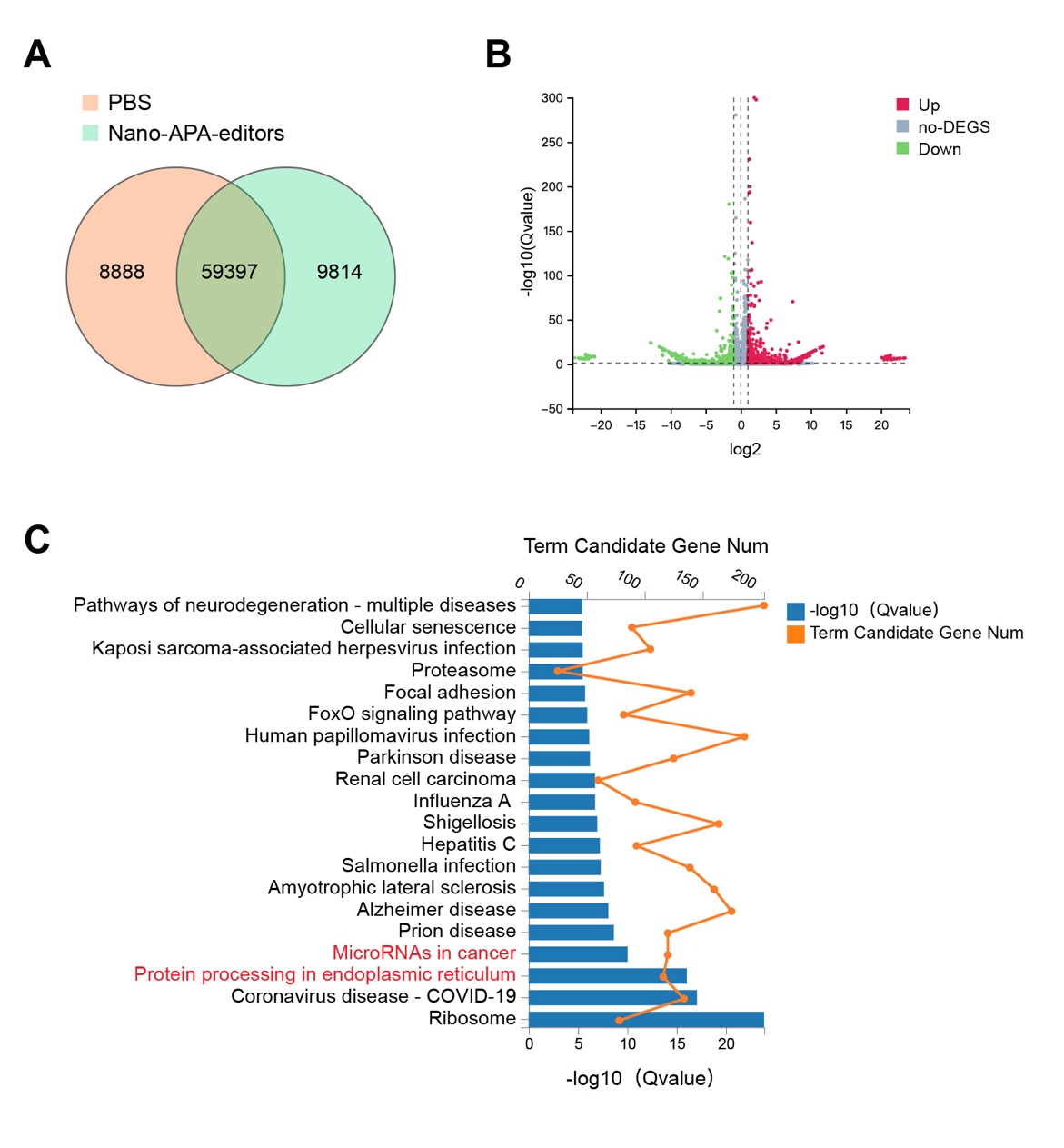


**Figure S6. Parallel analysis of** **differentially expressed transcripts.** A) A Venn diagram revealed the number of transcripts transcribed in each treatment group. B) Volcano plot and C) KEGG pathway analysis of differentially expressed transcripts activated by Nano-APA-editors.


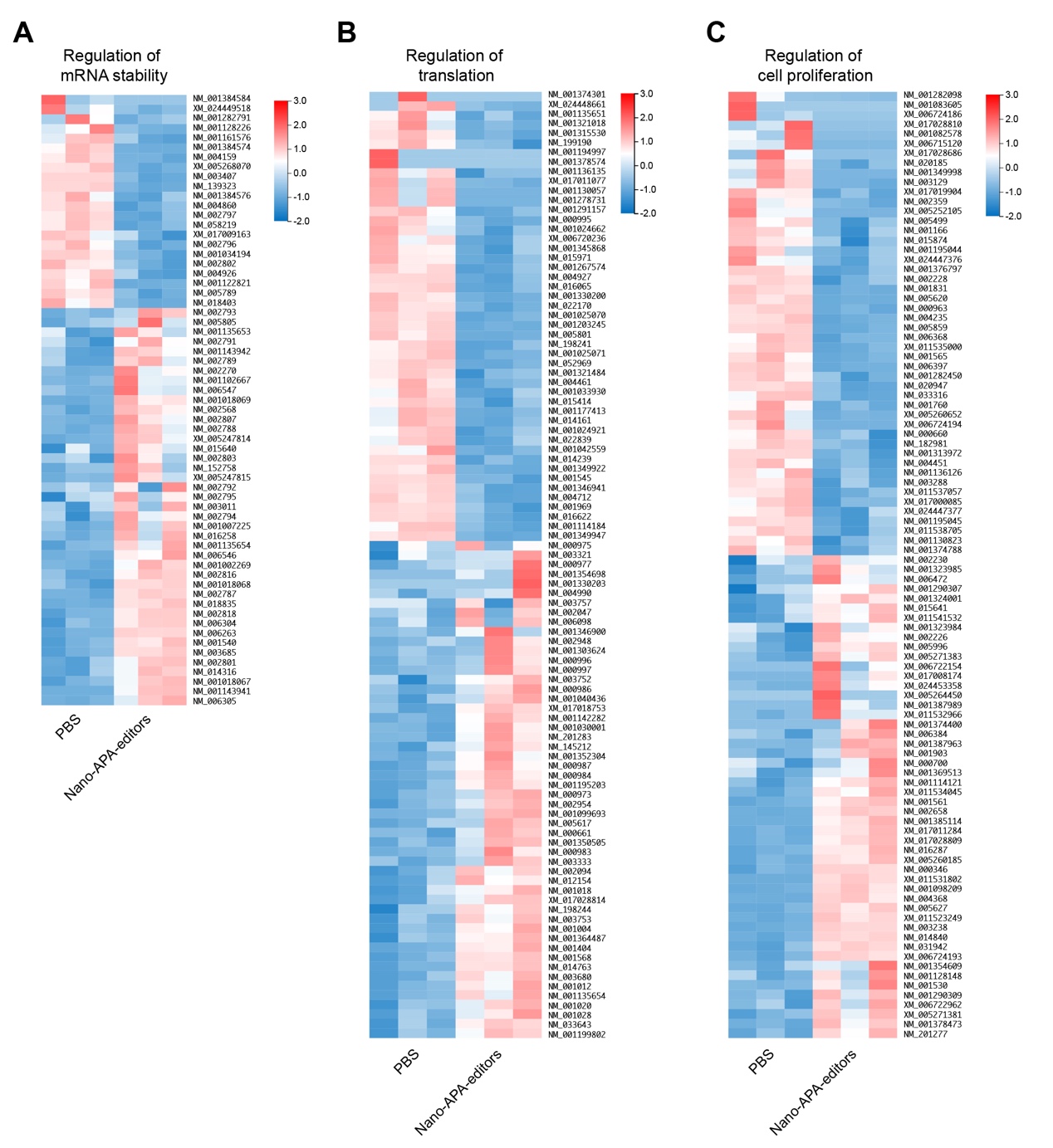


**Figure S7. Heatmap of transcripts, which were associated with regulation of A) mRNA stability, B) translation and C) cell proliferation.**


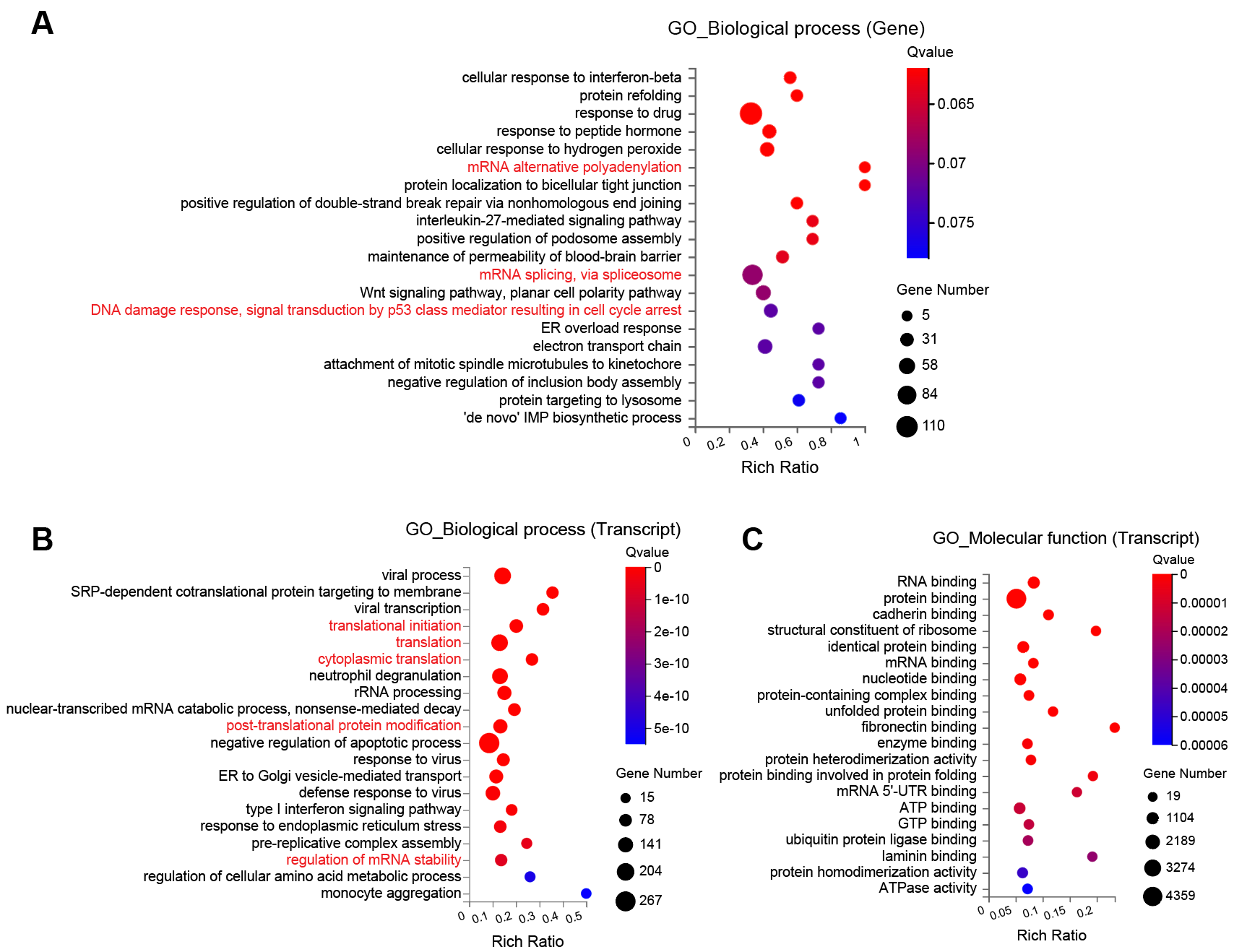


**Figure S8. GO enrichment analysis of differential genes and transcripts.** A) Represents GO enrichment analysis for biological processes in DEGs and B) DETs. C) Displays GO enrichment for molecular functions in DETs.


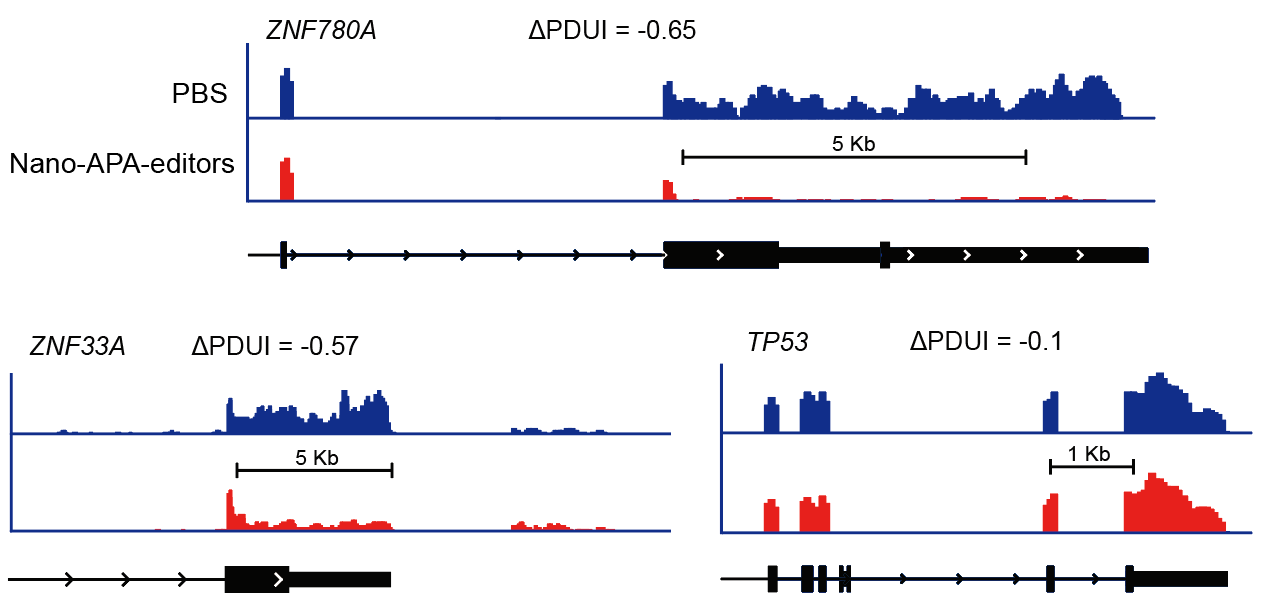


**Figure S9. Representative RNA-seq density plots along with ΔPDUI values for genes.**


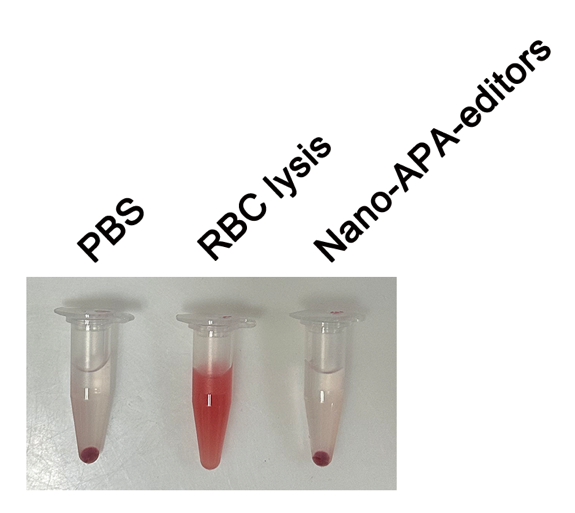


**Figure S10. Photos of the nanomaterial hemolysis test.**

*Materials*: Cetyltrimethylammonium bromide (CTAB), tetraethyl orthosilicate (TEOS), triethanolamine (TEA), and cholesterol (Chol) were purchased from Aladdin Reagent (Shanghai, China). Penicillin, streptomycin, and Dulbecco's modified Eagle's medium (DMEM) were purchased from Thermo Fisher Scientific (USA). Fetal bovine serum (FBS) was purchased from Biological Industries (Israeli). PEI was purchased from Polysciences (USA). Anti-CFIm25 antibody (10322-1-AP), PTEN (60300-3-Ig) and anti-secondary antibody (RGAR001) were purchased from Proteintech (China). Anti-TGF-β (PH07319S) and WEE1 (PU323164S) antibodies were purchased from Abmart (China). Rabbit anti-Ki67 (ab16667) antibody was purchased from Abcam (UK).

*Oligo:* NUDT21, sense: 5'- TTGCATTGGTAACTGGTGGA-3'; antisense: 5'- GATGGGTCCATATCCTGGTG-3'; GAPDH, sense: 5'- CTGGGCTACACTGAGCACC-3'; antisense: 5'- AAGTGGTCGTTGAGGGCAATG-3'; PTEN-proximal, sense: 5'- ACTGCTTGTTGTTTGCGCAT-3'; antisense: 5'- GCCCATTCTTTGTTGATAGCCT-3'; PTEN-distal, sense: 5'- CACCTGGAGACATAGCCATCG-3'; antisense: 5'- TCACTGTCCGTTTTCTGATGC-3'; TGFB2-proximal, sense: 5'- GCAATAGGCACCCTTCCCAT-3'; antisense: 5'- AATGCAGACTTTCTCGGTCA-3'; TGFB2-distal, sense: 5'- TTGCCCTCTGTGCTTTCTCC-3'; antisense: 5'- TGGCGTTGGCAGAACATAGA-3'; WEE1-proximal, sense: 5'- TACTCCTTTCCCACCTCCCC-3'; antisense: 5'- TAACCCTGGAAAGGCTGGTG-3'; WEE1-distal, sense: 5'- TGTTTTGCCCGGTTTTTCTCT-3'; antisense: 5'-ACAAGTCAAAGACAAGTGCAAACA-3'; Aptamer TA Sequence: 5'-chol-TGTGGTTGGCTTCGTGTGCGCGTGTGGGTG-3’ were obtained Sangon Biotechnology.

*Preparation of sgRNA:* The sgRNA sequence targeting the human NUDT21 gene was designed and rigorously screened using the CHOPCHOP online algorithm (GRCh38/hg38 assembly). To ensure optimal on-target cleavage efficiency and minimize off-target potential, the selected 20-bp target sequence (5'-AGCTGCAGCAGTAACACATG-3') was specifically chosen for its ideal GC content (50%), high predicted cleavage efficiency (score: 69.24), and zero predicted off-target sites with up to two base mismatches (MM0 = 0, MM1 = 0, MM2 = 0) across the entire human genome. A double-stranded DNA (dsDNA) template for in vitro transcription (IVT) was generated by PCR using the following full template sequence, which includes the T7 RNA polymerase promoter (underlined), the NUDT21-specific target sequence (in bold), and the sgRNA scaffold backbone (in italics): 5'-TTCTAATACGACTCACTATAAGCTGCAGCAGTAACACATGGTTTTAGAGCTAGA-3'*.* A non-targeting scramble control sgRNA (sgScr) was prepared using a similar method with a non-homologous sequence 5'-TTCTAATACGACTCACTATAGGTACAGTCAGAGCCAACCTGTTTTAGAGCTAGA-3'. The sgRNAs (both sgNUDT21 and sgScr) were subsequently synthesized using a Hifair Precision sgRNA Synthesis Kit (Yeasen, Shanghai, China) according to the manufacturer’s protocol. The resulting sgRNAs were purified using an RNA purification kit (Tiangen, Beijing, China), quantified using a NanoDrop spectrophotometer, and their integrity was confirmed by RNA denaturing gel electrophoresis.

*Assembly of Cas9 Ribonucleoprotein (RNP) Complex:* Cas9 protein and sgRNA were mixed in equal volumes and incubated at room temperature (25°C) for 1 hour to allow for the complete self-assembly of the RNP complex. For the control RNP complex, Cas9 protein was assembled with the sgScr using an identical procedure.

*In Vitro Cleavage Assay (RNP Activity Validation):* To validate the cleavage activity of the assembled IRCas9 RNP, an in vitro digestion assay was performed.

First, the genomic DNA (gDNA) was extracted from SCC25 cells using a Cell DNA Extraction Kit (Tiangen, Beijing, China). The target region of the NUDT21 gene was amplified from the gDNA by PCR, yielding the product. The primers used for amplification were: NUDT21, sense: 5'-GTGGAATGCAGGGGGACATT-3'; antisense: 5'-GCCTGTCTGCTGGACTAAGG-3'

The purified NUDT21 PCR product (200 ng) was then incubated with the pre-assembled IRCas9 RNP complex (100 nM) in a 1× cleavage buffer (NEBuffer) in a total volume of 20 μl. The reaction mixture was incubated at 37°C for 30 min on a thermomixer. The reaction was subsequently terminated by adding 6× DNA Loading Dye containing SDS. The cleavage products were resolved by 1% agarose gel electrophoresis and visualized.

*Nucleic Acid Leakage Rate and Realease Studies:* To evaluate the stability of the nanocomplexes, HMSNs-PEI@IRCas9 and HMSNs-PEI@IRCas9@M were stored at 4 °C and sampled on day 0, day 1, day 3, day 5 and day 7. At each time point, the samples were centrifuged (10,000 × g, 10 min), and the supernatants were collected to quantify the released sgRNA by measuring the absorbance at 260 nm using a NanoDrop ultra-micro spectrophotometer (Thermo Scientific). The nucleic acid leakage rate was calculated as the percentage of RNA released relative to the total RNA loaded.

For the in vitro release assay, the nanocomplexes were incubated in simulated body fluid (SBF, pH 7.4) at 37 °C under gentle shaking. At predetermined time points, the supernatant was collected after centrifugation and replaced with an equal volume of fresh SBF. The released sgRNA was quantified by NanoDrop spectrophotometry, and cumulative release profiles were plotted as a function of incubation time.

*Agarose Gel Electrophoresis Assay:* Agarose gel electrophoresis was performed to evaluate the binding and protection efficiency of nucleic acids in the nanocomplexes. The nanoparticles were loaded onto a 1.0 wt% agarose gel containing GelRed nucleic acid dye and electrophoresed at 60 V for 40 min in 1× TBE buffer. The migration of free and complexed sgRNA or Cas9 bands was visualized using a Bio-Rad Gel Doc XR+ imaging system.

*Bioinformatics Analysis:* The expression pattern of NUDT21 in HNSC and normal tissues was analyzed using the GEPIA (Gene Expression Profiling Interactive Analysis, http://gepia.cancer-pku.cn) online database, based on data from the TCGA and GTEx projects. The survival analyses of NUDT21 and other APA-related core factors were performed using the “Survival” module of GEPIA, where patients were divided into high- and low-expression groups according to median gene expression levels.

The association between NUDT21 expression and clinical stage of HNSC was evaluated using the “Stage Plot” function in GEPIA. In addition, correlation analyses between NUDT21 and key tumor suppressor genes (PTEN, TP53, and RB1) were carried out using Pearson correlation within the same platform.

*Cell Viability Assay:* The cytotoxicity of the nanomaterials toward SCC25 cells was evaluated using a Cell Counting Kit-8 (CCK-8, Solarbio, China). SCC25 cells were seeded in 96-well plates at a density of 5 × 10³ cells per well and allowed to adhere overnight. The cells were then treated with various concentrations of PBS, HMSNs and Nano-APA-editors for 24 h. After incubation, 10 µL of CCK-8 solution was added to each well, followed by a 2 h incubation at 37 °C. The absorbance was measured at 450 nm using a microplate reader (BioTek Synergy HTX, USA).

*Quantitative PCR Analysis:* SCC25 cells were washed twice with PBS, and approximately 5 × 10⁴ cells were lysed in 800 μL of TRIzol reagent (Thermo Fisher Scientific) for 5 min at room temperature. Chloroform was added, and the mixture was incubated for 5 min at room temperature, followed by centrifugation at 15,000 rpm for 20 min at 4 °C. The aqueous phase was collected, and total RNA was precipitated with an equal volume of isopropanol, washed twice with 75% ethanol, air-dried, and dissolved in RNase-free water.

Complementary DNA (cDNA) was synthesized using the All-In-One 5× RT MasterMix (Applied Biological Materials). Quantitative real-time PCR (qRT-PCR) was performed using Power SYBR™ Green PCR Master Mix (Thermo Fisher Scientific) on a QuantStudio™ 1 Real-Time PCR System according to the manufacturer’s instructions. Relative mRNA expression levels were calculated using the 2⁻ΔΔCt method, normalized to GAPDH as an internal reference.

*Colony Formation Assay:* SCC25 cells were seeded in 6-well plates and treated with PBS, HMSNs, HMSNs-PEI@IR(Scr), HMSNs-PEI@IRCas9, and Nano-APA-editors for 24 h. After treatment, 500 viable cells per well were reseeded in DMEM supplemented with 20% FBS and cultured for 7 days. The colonies were washed twice with PBS, fixed with 4% paraformaldehyde for 20 min, and stained with 0.1% crystal violet for 30 min.

*Transwell Migration and Invasion Assays:* Transwell assays were performed to evaluate the migration and invasion abilities of tumor cells. For the migration assay, SCC25 cells were seeded in 6-well plates and treated with PBS, HMSNs, HMSNs-PEI@IR(Scr), HMSNs-PEI@IRCas9, and Nano-APA-editors. After 24 h, 24-well plates containing 20% FBS in DMEM were assembled with Transwell inserts (8 μm pore size, Corning Falcon). The upper chamber was filled with 200 μL serum-free DMEM containing 2 × 10⁵ cells, and the cells were incubated for 24 h at 37 °C. Non-migrated cells on the upper surface of the membrane were removed with a cotton swab, while migrated cells on the lower surface were fixed and stained with 0.1% crystal violet for 10 min. The inserts were washed with PBS, and migrated cells were photographed and counted in three randomly selected fields per insert.

For the invasion assay, 15 μL of Matrigel (ABW Matrigengel) was precoated on the upper surface of the inserts and allowed to solidify at 37 °C for 1 h. Subsequent steps were performed as described for the migration assay.

*Wound-Healing Assay:* SCC25 cells were seeded in 6-well plates and incubated overnight to allow cell attachment. The cells were then treated with PBS, HMSNs, HMSNs-PEI@IR(Scr), HMSNs-PEI@IRCas9, and Nano-APA-editors. A straight scratch was generated in the cell monolayer using a sterile 10 μL pipette tip. Detached cells were removed by washing twice with PBS, and the remaining cells were cultured in fresh medium. Images of the wound area were captured at 0 h and 12 h under a microscope (×40 magnification). The migration capacity was quantified by measuring the wound closure distance between the two time points.

*EdU Proliferation Assay:* Cell proliferation was assessed using a 5-ethynyl-2′-deoxyuridine (EdU) incorporation assay. SCC25 cells were seeded in 24-well plates and cultured overnight to allow cell attachment. The cells were then treated with PBS, HMSNs, HMSNs-PEI@IR(Scr), HMSNs-PEI@IR(Cas9), or Nano-APA-editors for 24 h at 37 °C. Subsequently, cells were incubated with EdU solution (10 μM, Beyotime, China) for 2 h according to the manufacturer’s protocol.

After incubation, the cells were fixed with 4% paraformaldehyde for 15 min at room temperature and permeabilized with 0.3% Triton X-100 for 15 min. The incorporated EdU was detected using a Click-iT reaction cocktail (Beyotime, China) containing fluorescent azide. Nuclei were counterstained with Hoechst 33342 (5 μg/mL) for 10 min. Images were captured using a fluorescence microscope (Leica DMi8), and the percentage of EdU-positive cells was calculated from five randomly selected fields per group.

*RNA Sequencing and Alternative Polyadenylation (APA) Analysis:* SCC25 cells (1 × 10⁶) were seeded in 6-well plates and cultured for 12 h, followed by treatment with PBS or Nano-APA-editors at 37 °C for 24 h. The experiments were performed using three independent biological replicates per group (n = 3). Total RNA was extracted using TRIzol reagent according to the manufacturer’s instructions. RNA integrity and concentration were assessed using an Agilent 2100 Bioanalyzer (Agilent Technologies).

RNA sequencing and library construction were performed by BGI (Shenzhen, China). On an Illumina platform, yielding an average sequencing depth of approximately 30 million clean reads per sample. Raw reads were quality-checked, trimmed, and aligned to the human reference genome (GRCh38) using HISAT2. For the estimation of relative gene expression levels, read counts were normalized to the FPKM (Fragments Per Kilobase of transcript per Million mapped reads) metric. Differential gene expression analysis was performed on raw read counts using the DESeq2 package, which incorporates its own internal median of ratios normalization method. Significantly differentially expressed genes (DEGs) were strictly defined as those with an adjusted *p* value (FDR) < 0.05 and ∣log_2_FC∣ ≥ 1. Gene Ontology (GO) and Kyoto Encyclopedia of Genes and Genomes (KEGG) enrichment analyses were subsequently conducted, and results were visualized using the Dr. Tom platform (BGI, Shenzhen, China).

APA profiling was performed using DaPars2 to quantify the percentage of distal poly(A) site usage index (PDUI) for each gene. Changes in APA usage between groups were represented as ΔPDUI values, and genes with |ΔPDUI| ≥ 0.2 and *p* < 0.05 were defined as significantly altered APA events.

*Public RNA-seq data processing and IGV visualization:* To validate the clinical relevance of APA events, bulk RNA-seq datasets of patient cohorts were retrieved from the GEO database. Raw sequencing reads (FASTQ format) were downloaded using the SRA Toolkit. After quality control and adapter trimming, the clean reads were mapped to the human reference genome (GRCh38/hg38) using HISAT2. To visualize the read coverage across specific genomic loci, the aligned BAM files were sorted and indexed using SAMtools. Subsequently, the BAM files were converted into continuous bigWig (.bw) format using the bamCoverage function within the deepTools suite. the read counts were normalized using the Counts Per Million (CPM) method to ensure accurate visual comparison of read densities across different samples and varying sequencing depths. The resulting bigWig files were then loaded into the IGV (Broad Institute, MA, USA), and the read coverage tracks spanning the *PTEN* coding sequence and its extended 3'UTR region were exported for comparative analysis.

*Establishment and Analysis of Orthotopic Tongue Tumor Model:* To establish the orthotopic tongue squamous cell carcinoma (OSCC) model, SCC25 cells (cells suspended in 30 μL of PBS was carefully injected into the lateral border of the tongue of anesthetized mice using a micro-syringe. When the orthotopic tongue tumors reached an average volume of approximately 30 mm, the mice were randomly divided into 5 groups (n = 5 mice per group): PBS, HMSNs, HMSNs-PEI@IRCas9, PTX and Nano-APA-editors. Treatments were administered via intratumoral injection at a dose of 5 mg/kg per mouse. The injections were given continuously for 3 days. Mice were monitored every 2 days for general health and tumor development. At the endpoint of the experiment day 21, all mice were ethically sacrificed, and the tongues were excised for imaging and analysis.

Photographs of the tumor-bearing mice and isolated tumor tissues were taken using a digital camera. Tumor weight and body weight were measured and recorded. The relative tumor burden was calculated as the ratio of tumor weight to total body weight. All animal procedures were performed in accordance with the institutional guidelines for animal care and approved by the Animal Ethics Committee.

*Histology and Hematoxylin–Eosin (H&E) Staining:* After sacrifice, tumor tissues and major organs (heart, liver, spleen, lung, and kidney) were excised, rinsed in PBS, and fixed in 10% neutral-buffered formalin for 24–48 h at room temperature. Fixed samples were dehydrated through a graded ethanol series, cleared in xylene, and embedded in paraffin. Sections (4 μm) were prepared using a microtome and mounted on glass slides. Paraffin sections were deparaffinized in xylene, rehydrated through descending ethanol concentrations to distilled water, and subjected to standard hematoxylin–eosin staining (hematoxylin for 5–10 seconds, rinsing in running water; eosin for 5–10 seconds). Slides were then dehydrated through ascending ethanol series, cleared in xylene, and coverslipped with a permanent mounting medium. Stained sections were imaged with a light microscope (Leica) and evaluated for histopathological changes. All slides were reviewed in a blinded fashion by a pathologist; representative images were captured and, where applicable, semi-quantitative scoring of tissue damage or inflammation was performed.

*Immunofluorescence Staining for Ki67:* Paraffin-embedded tumor sections were deparaffinized in xylene and rehydrated through graded ethanol to distilled water. Antigen retrieval was performed in 10 mM sodium citrate buffer (pH 6.0) at 95 °C for 15 min. After cooling, sections were permeabilized with 0.3% Triton X-100 for 10 min and blocked with 5% bovine serum albumin (BSA) for 30 min at room temperature. Sections were then incubated overnight at 4 °C with anti-Ki67 primary antibody (1:200 dilution) diluted in blocking buffer. After washing with PBS, samples were incubated with Alexa Fluor–conjugated secondary antibody (Invitrogen, 1:500 dilution) for 1 h in the dark at room temperature. Nuclei were counterstained with DAPI (1 μg/mL) for 10 min and mounted with antifade mounting medium.

Images were captured under a fluorescence microscope (Leica DM6 B), and five random high-power fields were analyzed for each sample. The Ki67 index was calculated as the percentage of Ki67–positive nuclei relative to total DAPI-stained nuclei using ImageJ (FIJI) software.

*TUNEL Assay:* Apoptotic cells in tumor tissues were detected using a TUNEL assay kit (Beyotime, C1086) following the manufacturer’s protocol. Briefly, paraffin-embedded sections were deparaffinized, rehydrated, and permeabilized with 0.3% Triton X-100 for 10 min. After rinsing, sections were incubated with TUNEL reaction mixture at 37 °C for 1 h in a humidified chamber. Negative controls were treated without terminal deoxynucleotidyl transferase, and positive controls were pretreated with DNase I for 10 min.

After incubation, nuclei were counterstained with DAPI and mounted with antifade mounting medium. Fluorescence images were acquired using the same exposure parameters for all samples. The apoptotic index was quantified as the percentage of TUNEL-positive nuclei in five random fields (400×) per tumor section using ImageJ.

*Immunohistochemistry (IHC):* Paraffin-embedded tumor sections were deparaffinized in xylene and rehydrated through a graded ethanol series to distilled water. Endogenous peroxidase activity was blocked by incubation in 3% hydrogen peroxide in methanol for 10 min. Antigen retrieval was performed by heating sections in 10 mM sodium citrate buffer (pH 6.0) or Tris-EDTA buffer (pH 9.0) (choose buffer according to antibody datasheet) at 95–100 °C for 15–20 min. After cooling to room temperature, sections were washed in PBS and blocked with 5% normal goat serum (or 5% BSA) for 30 min at room temperature.

Sections were incubated overnight at 4 °C with primary antibodies (1:100–1:400). After three washes in PBS, slides were incubated with an appropriate HRP-conjugated secondary antibody for 30–60 min at room temperature. Immunoreactivity was visualized using 3,3′-diaminobenzidine (DAB) substrate until desired signal developed, followed by rinsing in tap water. Sections were counterstained with hematoxylin, dehydrated through graded ethanol, cleared in xylene, and coverslipped with a permanent mounting medium. Images were acquired using a bright-field microscope with identical exposure and imaging settings across groups.

*Hemolysis Test:* 100 μl of 2% red blood cells were diluted to 1 ml with PBS, and 5 mg of Nano-APA-editors ware added. Incubate for 30 minutes in a 37 ℃ incubator. After centrifugation at 2000 g for 5 minutes, observing the hemolysis. A positive control was prepared by treating RBC with ACK Lysis Buffer (Thermo Fisher Scientific).
